# Interplanting potato with grapes improved yield and soil nutrients by optimizing the interactions of soil microorganisms and metabolites

**DOI:** 10.3389/fpls.2024.1404589

**Published:** 2024-09-09

**Authors:** Chengchen Li, Yuming Xie, Yongshan Liao, Jitao Liu, Bin Li, Yusheng Lu, Kun Yang, Jianwei Shan, Li Wang, Kang An, Xiaoqi Zhou, Xu Cheng, Xiaobo Li

**Affiliations:** ^1^ Guangdong Provincial Key Laboratory of Crops Genetics and Improvement, Crop Research Institute, Guangdong Academy of Agriculture Sciences, Guangzhou, China; ^2^ Institute of Facility Agriculture, Guangdong Academy of Agriculture Sciences, Guangzhou, China; ^3^ Institute of Agricultural Resources and Environment, Guangdong Academy of Agricultural Sciences, Guangzhou, China; ^4^ School of Ecology and Environmental Science, East China Normal University, Shanghai, China; ^5^ Guangdong Laboratory of Lingnan Modern Agriculture, Key Laboratory of Synthetic Biology, Ministry of Agriculture and Rural Affairs, Agricultural Genomics Institute at Shenzhen, Chinese Academy of Agricultural Sciences, Shenzhen, China

**Keywords:** bacterial community, metabolites, 13-L-hydroperoxylinoleic acid, variety, enzyme activity

## Abstract

Interplanting crops is the best method to grow crops synergistically for better utilization of land and agro-resources. Grape (*Vitis vinifera*) and potato (*Solanum tuberosum* L.) have highly efficient agricultural planting systems in China, however, how soil physicochemical properties and soil microbial communities and metabolites affect the output of grape-potato interplanting remained unknown. In this study, we employed three planting patterns (CK: grape monocropping; YY: grape interplanted with potato (variety ‘*Favorita*’); LS: grape interplanted with potato (variety ‘*Longshu7*’)) at two experimental sites i.e., the Huizhou (2022) site and the Qingyuan site (2023). The grape variety for all planting patterns was ‘*Sunshine Rose*’. Soil samples (top 0-20 cm) at both sites were collected to observe the diversity of bacterial communities and soil metabolites. Our findings revealed that, compared with monocropping, the interplanted systems resulted in higher concentrations of total nitrogen, available phosphorus, and available potassium and enhanced the activities of acid phosphatase, urease, and protease. The potato root exudates also altered the relative abundance of *Bacillus*, *Kaistobacter*, and *Streptomyces* in the rhizosphere. Among the soil metabolites, lipids and organic acids showed the most significant changes. Notably, 13-L-hydroperoxylinoleic acid is the key differentially abundant metabolite involved in the regulation of linoleic acid metabolism pathways. The association analyses of the metabolome, microbiome, and soil physicochemical properties revealed that the interactions of microbes and metabolites resulted in differences in the soil nutrient content, whereas the interactions of 13-L-hydroperoxylinoleic acid and *Firmicutes* improved the soil nutrient levels and bacterial composition in the interplanting systems. In summary, our findings demonstrated that intercropping grapes with potato ‘*Favorita*’ was better with respect to improving soil nutrients, soil enzyme activity, the diversity of soil bacteria, and soil metabolites without causing adverse impacts on grape yield. Overall, this study explained the physiological mechanisms by which soil microorganisms and metabolites promote potato growth in grape interplanting and provided new perspectives for the utilization of soil resources in vineyards.

## 1 Introduction

Fruits, cereals, and vegetables are important agricultural crops and are widely cultivated in China. Interplanting is a special form of planting system with complementary utilization of resources, time, and space to grow crops synergistically that substantially reduces the chances of pests and diseases and enhances the economic benefits compared with traditional monocultures (Li et al., 2018; Qiao et al., 2019; Chang et al., 2022; Duan et al., 2023; Wang et al., 2023). Interplantation of potato (*Solanum tuberosum* L.) with grapes (*Vitis vinifera*) is advantageous as the dormant period of grapevines can be better utilized with respect to spatiotemporal variability. Interplanting for complementary utilization of natural resources not only enhances total output but also increases the orchard replanting index and overall planting intensity which is favorable for the sustainable production of potatoes and grapes (Qiao et al., 2019; Li et al., 2022; Tang et al., 2022).

The grape is one of the major fruits in China that can be intercropped with multiple crops including Bidens species, tobacco, tall fescue, and vegetables (Celette et al., 2005; Wang et al., 2015; Xia et al., 2018). The intercropping of grape seedlings with Bidens improved the soil organic matter content and increased the activities of urease, invertase, and catalase in soil under Cd toxicity (Xia et al., 2018). Intercropping grapes with tobacco effectively controlled grape phylloxera (a worldwide grapevines pest). Intercropping grapes with tall fescue can limit water competition (Celette et al., 2005). Intercropping potato-onion and tomatoes enhanced the soil organic carbon content (Li et al., 2020).

Potato is the fourth largest staple food in China (Wang Q. et al., 2023). In previous studies, grape−potato intercropping has focused on cultivation management techniques for interplanting (Chen et al., 2019; De Conti et al., 2019), however, there is a lack of research on the effects of interplanting different varieties of potatoes and grapes on soil bacterial communities and soil metabolites.

Soil metabolites originate from various sources, including plant roots and soil microorganisms during the decomposition of organic matter (Deng et al., 2021; Mahmood et al., 2023; Shi et al., 2024). The plant root system recruits beneficial soil microorganisms to colonize the vicinity of the roots through the release of metabolite-signaling substances that facilitate nutrient decomposition and turnover in the soil for optimal plant growth (Wang et al., 2017, Wang S. et al., 2023). Conversely, changes in soil structure and microbial communities affect the diversity, composition and concentration of plant metabolites in the soil (Sun L. et al., 2022; Duan et al., 2023; Wang et al., 2023). For example, intercropping tea plants [*Camellia sinensis* (L.) O. Kuntze] with mung bean or adzuki bean improved the soil nutrients, organic matter, and soil organic carbon (Wang S. et al., 2023). Purine metabolism in sugarcane-peanut intercropping promoted the secretion and accumulation of adenosine and adenine in roots and rhizosphere soils, increased the total phosphorus and potassium contents, increased acid phosphatase and urease activities, and improved rhizosphere soil physicochemical properties (Tang et al., 2022). Furthermore, the selection of crop species and intercropping patterns has an impact on the changes in soil nutrient status, the microbial community, and metabolites (Tang et al., 2022; Duan et al., 2023). Although the potential benefits of intercropping grapes with potatoes are well recognized, however, there is a lack of understanding regarding the impact of grape−potato interplanting on soil microbial communities and metabolites.

Compared with monocropping, the crop yields from interplanting are largely affected by the utilization of plant nutrition (Wang et al., 2016; Tang et al., 2022; Secco et al., 2023; Shen et al., 2023; Wang W. et al., 2023; Zhu et al., 2023). Wang et al. (2021) observed marginal variations in grape yield within interplanting systems (Wang et al., 2021). Consequently, we hypothesized that microorganisms in the rhizosphere soil of grapes and potatoes do not compete for resources but reciprocally benefit each other. Therefore, in this study, we examined the effects of interplanting grapes and potatoes in vineyards on overall productivity including soil nutrients, enzyme activities, bacterial community composition, and metabolite diversity, aiming to analyze the physiological mechanisms by which soil beneficial microorganisms and metabolites promote potato growth in grape-potato interplanting systems. This study would be helpful in order to specify the interplanting patterns of the widely-grown potato varieties in vineyards to improve overall system productivity and profitability.

## 2 Materials and methods

### 2.1 Experimental description

The experiment was conducted at the Longhua Vineyard (24°19′19″N, 113°20′44″E) in Qingyuan (QY) City from 15 November 2023 to 28 February 2024, and at the Ink Garden Book Farm (23°13′30″N, 114°35′7.9″E) in Huizhou (HZ) City from 15 November 2022 to 26 February 2023 Guangdong Province, China. The climate type is classified as subtropical humid, with an average temperature of 20.2°C in QY and 21.1°C in HZ during the growing period. The soils at the HZ and QY sites are classified as Ferralisols with a medium loam texture (Zhang et al., 2014; Wang et al., 2022). The experimental sites were previously planted with grapes. One grape variety ‘*Sunshine Rose*’ and the two potato varieties, i.e., ‘*Favorita*’ and ‘*Longshu7’* (resistant to late blight), were planted. The experimental treatments were comprised of three planting treatments i.e., CK: grape monocropping; YY: grape interplanted with potato (variety ‘*Favorita*’); LS: grape interplanted with potato (variety ‘*Longshu7*’). The experiment was set up in a randomized complete block design with four replicates, each with a size of 156 m^2^ (12 m long by 13 m wide). Grapes and potatoes were grown in greenhouses.

The soil was collected at a depth of 0–0.2 m below the surface. The rhizosphere soil mixtures were collected from three points of potato growth in each replicate. Four replicate samples were collected from each treatment. A total of 12 soil samples were collected and sieved through a 20-mesh sieve to remove plant roots, animal remains, and other impurities and then divided into three portions and each portion was used to determine soil properties, soil enzyme activities, and sequencing of the microbiome (16S) and metabolome (Liquid chromatography-mass spectrometry (LC-MS)) at the Suzhou Panomix Biomedical Tech Co., Ltd. The soil chemical properties at HZ and QY were as follows: pH: 7.28 and 7.33; electrical conductivity: 0.16 and 0.11 mS/cm; available phosphorus: 143.79 and 129.79 mg/kg; available potassium: 845.03 and 545.03 mg/kg; and organic matter: 32.67 and 27.67 g/kg. The monoculture and interplanting arrangements in vineyards were as follows: potatoes and grapes were planted at a distance of 0.85 m, and the grapes were planted at a row spacing of 2.6 m, with an aisle of 0.6 m in the middle. The potatoes were planted in single rows with 0.25 m plant spacing (**Supplementary Figure S1**). The yields were measured, the soil samples were collected at harvest, and the same fertilizer rates and management practices were applied during planting at the HZ and QY sites.

### 2.2 Determination of soil properties and enzyme activities

The soil properties were determined with reference to previously reported methods and improvements (Bao, 1999; Wang S. et al., 2023). The soil pH and electrical conductivity were determined with a glass electrode after 10 g of soil was suspended in 25 mL of deionized water (1:2.5, w/v) (Li et al., 2020). The total phosphorus (TP) content was determined by digesting 1 g of soil in H_2_SO_4_-HClO_4_ via the molybdenum-antimony colorimetric method (Wang et al., 2022). The total potassium (TK) content was determined via the flame photometric method (ZA-3300, Hitachi, Japan) by melting 1 g of soil and 2 g of NaOH (Wang et al., 2022). Total nitrogen (TN, including NH_4_^+^-N and NO_3_^−^-N) was determined via the Kjeldahl method via wet burning of 1 g of soil and 2 mL of H_2_SO_4_ (Bao, 1999; Wang et al., 2022). Ammonia nitrogen (NH_4_-N) and nitrate nitrogen (NO_3_-N) were extracted with 0.5 g of CaSO_4_·2H_2_O and 2 M KCl, respectively, and then analyzed with a continuous flow analyzer (San++, SKALAR, Netherlands) (Bao, 1999; Li et al., 2020). Available phosphorus (AP) was extracted from 2.5 g of soil and 0.5 M NaHCO_3_ and analyzed via the ammonium molybdate method (Bao, 1999; Li et al., 2020). Available potassium (AK) was extracted from 5 g of soil and 1.0 M NH_4_Ac and then quantified via the flame photometric method (ZA-3300, Hitachi, Japan) (Bao, 1999; Li et al., 2020). Organic matter (OM) and organic carbon (OC) were extracted via K_2_Cr_2_O_7_ and H_2_SO_4_ to digest 0.5 g of soil, and the residual K_2_Cr_2_O_7_ was titrated with 0.2 M FeSO_4_·7H_2_O (Bao, 1999; Li et al., 2020; Wang et al., 2022). The activities of the soil enzymes were determined via a kit from Solarbio^®^ Science & Technology Co. (Beijing, China). Phosphatase activity was measured via the phenol colorimetric method, protease activity was assessed via the tungsten blue method, urease activity was quantified via the indophenol blue colorimetric method, and catalase and neutral converting enzyme activities were analyzed via a characteristic light absorption method (Hou et al., 2020).

### 2.3 DNA extraction from soil and construction of the sequencing library

The DNA from soil samples (0.5 g) was extracted via the EZNA^®^ Soil DNA Kit (Omega Biotek, Inc., Norcross, GA, USA) and quantified via Nanodrop. The quality of the extracted DNA was detected via 1.2% agarose gel electrophoresis (Wang S. et al., 2023). The Pfu high-fidelity DNA polymerase was obtained from TransGen^®^, and the forward primer 338F (5’-ACTCCTACGGGGAGGCAGCA-3’) and the reverse primer 806R (5’-GGACTACHVGGGGTWTCTAAT-3’) were used for PCR amplification of the extracted soil DNA. The amplified recovered products were quantified by fluorescence using the Quant-iT PicoGreen dsDNA assay kit in an instrument microplate reader (BioTek, FLx800), and the samples were mixed according to the corresponding proportions according to the amount of sequencing volume required for each sample. The sequencing library was constructed via Illumina’s TruSeq Nano DNA LT Library Prep Kit. Before sequencing, the libraries were quality checked and quantified, and the qualified online sequencing libraries were gradient diluted, mixed according to the corresponding ratio, denatured to single-stranded by NaOH, and sequenced via a PE250 with an Illumina NovaSeq 6000 instrument.

### 2.4 Analysis of soil microbial sequencing data

The raw downstream data from high-throughput sequencing were initially screened according to sequence quality, and problematic samples were retested and retrosequenced. The raw sequences that passed the initial quality screening were divided into libraries and sampled according to index and barcode information, with subsequent removal of the barcode. Sequence denoising ASV clustering was performed according to the QIIME2 dada2 analysis process or the analysis process of V search software. The specific composition of each sample (group) at different species’ taxonomic levels was determined, the alpha diversity level of each sample was assessed according to the distribution of “ASV” in different samples, and the distance matrix of each sample was calculated to measure the difference in beta diversity and the significance of the difference among different treatments. Association networks were constructed, topological indices were calculated, key species were identified, the metabolic functions of colony samples were predicted, differential pathways were identified, and the species compositions of specific pathways were obtained.

### 2.5 Preparation of metabolomics samples

An appropriate amount of sample was accurately weighed into a 2 mL centrifuge tube, 600 µL of MeOH (containing 2-amino-3-(2-chloro-phenyl)-propionic acid (4 ppm)) was added, the mixture was vortexed for 30 s, steel balls were added, and the mixture was placed in a tissue grinder for 90 s at 55 Hz. The samples from the grinder were sonicated at room temperature for 30 min and then chilled on ice for 30 min. Supernatants were collected by centrifugation at 12000 rpm for 10 min at 4°C, filtered through a 0.22 μm membrane, and transferred to a detection vial for LC-MS analysis.

The LC analysis was performed on a Vanquish UHPLC System (Thermo Fisher Scientific, USA). Chromatography was carried out with an ACQUITY UPLC ^®^ HSS T3 (2.1×100 mm, 1.8 µm) (Waters, Milford, MA, USA). The column was maintained at 40°C. The flow rate and injection volume were set at 0.3 mL/min and 2 μL, respectively. For LC-ESI (+)-MS analysis, the mobile phases consisted of (B2) 0.1% formic acid in acetonitrile (v/v) and (A2) 0.1% formic acid in water (v/v). Separation was conducted with the following gradient: 0~1 min, 8% B2; 1~8 min, 8%~98% B2; 8~10 min, 98% B2; 10~10.1 min, 98%~8% B2; and 10.1~12 min, 8% B2. For LC-ESI (-)-MS analysis, the samples were analyzed with (B3) acetonitrile and (A3) ammonium formate (5 mM). Separation was conducted with the following gradient: 0~1 min, 8% B3; 1~8 min, 8%~98% B3; 8~10 min, 98% B3; 10~10.1 min, 98%~8% B3; and 10.1~12 min, 8% B3.

Mass spectrometric detection of metabolites was performed on an Orbitrap Exploris120 (Thermo Fisher Scientific, USA) with an ESI ion source. Simultaneous MS1 and MS/MS (full MS−ddMS2 mode, data-dependent MS/MS) acquisition was used with following adjustments: sheath gas pressure, 40 arb; aux gas flow, 10 arb; spray voltage, 3.50 kV and −2.50 kV for ESI(+) and ESI(−), respectively; capillary temperature, 325°C; MS1 range, m/z 100–1000; MS1 resolving power, 60000 FWHM; number of data-dependent scans per cycle, 4; MS/MS resolving power, 15000 FWHM; normalized collision energy, 30%; and dynamic exclusion time, automatic.

### 2.6 Analysis of metabolomic data

The raw mass spectrometry downstream files were converted to the mzXML file format via the MSConvert tool in the Proteowizard software package (v3.0.8789). Peak detection, peak filtering, and peak alignment were performed via the R XCMS software package to obtain a quantitative list of substances. The public databases HMDB, massBank, LipidMaps, mzcloud, KEGG, and self-constructed substance libraries were used for the identification of substances, and the LOESS signal correction method based on the QC samples was used to correct the data and eliminate systematic errors. Substances with RSDs > 30% in the QC samples were filtered out for data quality control.

The sample data were analyzed via partial least squares discriminant analysis (PLS-DA) downscaling via the R software package Ropls. On the basis of the statistical test used to calculate the P value, the OPLS-DA dimensionality reduction method to calculate the variable projection importance (VIP), and the fold change (FC) to calculate the multiplicity of difference between groups, we measured the intensity of the effect of the content of each metabolite component on the categorical discrimination of the samples and the explanatory ability, which assisted in the screening of marker metabolites. Pathway analysis was performed via the MetaboAnalyst software package for functional pathway enrichment and topology analysis of the screened differentially abundant metabolite molecules. The enriched pathways were used to browse the differentially abundant metabolite and pathway maps via the KEGG Mapper visualization tool. Venn diagrams, heatmaps, chord diagrams, and network diagrams used in the data analysis were plotted with the help of the Nomi Cloud tool.

### 2.7 Data analyses

The Kolmogorov–Smirnov and Levene methods were used in SPSS 19.0 to test the normal distribution and homogeneity of variance of the data (Che et al., 2024). One-way analysis of variance (ANOVA) with Duncan’s test via SPSS 19.0 (IBM SPSS Inc., USA) revealed statistically significant differences among treatments at *P* < 0.05 for soil properties, and Student’s t-test was used to compare yield and enzyme activities between the two treatments (Wang et al., 2022). The QIIME2 software (2019.4) and the Kruskal-Wallis test (the alpha value was 0.05) were used to calculate the significant differences in alpha and beta diversity, whereas species accumulation curves and rank abundance curves were generated via R project software via the ‘vegan’ R package at the OTU level (Li et al., 2020). The difference in OTU abundance was calculated via a Venn diagram via R project software in the ‘VennDiagram’ R package. The normalized heatmap data were compared via R project software via the ‘pheatmap’ R package with Euclidean distance and the Pearson coefficient method (*P* < 0.05). The linear discriminant analysis effect size (LEfSe) method ensures the selection of abundant bacteria that are significantly associated with monoculture and interplanting systems (Li et al., 2020).

Metabolite data were log_2_-transformed for statistical analysis and normalized (Tang et al., 2022). The metabolites and their correlations were calculated via R language (Wang S. et al., 2023). Partial least squares-discriminant analysis was used to calculate the significant differences in metabolite levels between different interplanting systems. A predictability Q2 value higher than 0.5 indicated better model fit accuracy, and a Q2 value close to 1 indicated greater reliability of the sample. Metabolite molecules were considered statistically significant when *P* was <0.05 and the VIP (variable importance in project) was >1. Finally, the KEGG database was used for pathway enrichment analysis of differentially abundant metabolites (Tang et al., 2022). Redundancy analysis (RDA) and the Mantel test were performed via the “vegan” package in R project software (version 4.2.0) to examine the relationships between the soil microbial communities and the soil properties (Li et al., 2020). Spearman rank correlations were calculated between metabolite VIP scores and different bacterial populations (Wang S. et al., 2023).

## 3 Results

### 3.1 Potato yield and soil physicochemical properties

**Figure 1:**
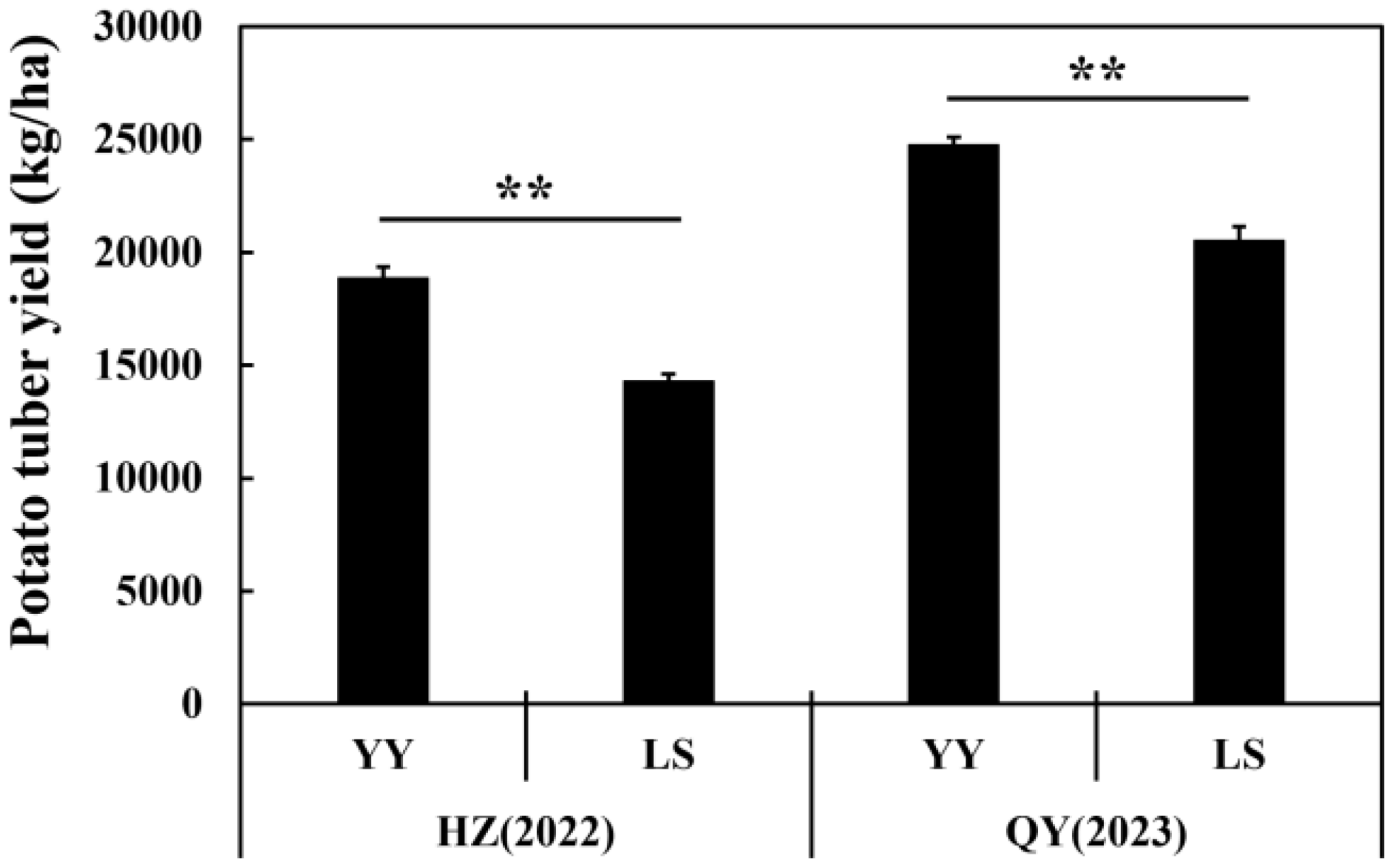
The potato tuber yield in different interplanting systems. The data for the two treatments were compared by Student’s t-test, and the error bars indicate the standard error of the mean with four biological replicates. ** represents 0.001≤ *P <*0.01 for YY compared with LS.

The potato tuber yield under the YY treatment was significantly (p < 0.01) greater than that under the LS treatment at both the HZ and QY sites (**Figure 1**). At the QY site, compared with the CK, interplanting with YY and LS significantly increased TN, AP, and AK 7.7% and 9.5%, 3.5% and 9.4%, and 20.7% and 6.3% respectively. However, it also led to a 2.8% and 3.2% reduction in the soil pH at the HZ and QY sites, respectively. Similarly, the grape-potato interplanting significantly reduced the contents of NH_4_-N, NO_3_-N, TP, and TK, which is consistent with the results observed at the HZ site (**Table 1**).

**Table 1:** The rhizosphere soil properties at the Houzhou and Qingyuan sites.

Site	Soil properties	CK	YY	LS
HZ (2022)	pH	7.28 ± 0.01a	7.16 ± 0.01b	7.12 ± 0.01c
	TN (g/kg)	1.45 ± 0.03b	1.61 ± 0.01a	1.64 ± 0.06a
	TP (g/kg)	1.40 ± 0.01a	1.33 ± 0.03a	1.16 ± 0.01b
	TK (g/kg)	12.35 ± 0.06a	10.65 ± 0.08b	9.21 ± 0.32c
	NH_4_-N (mg/kg)	16.80 ± 0.12a	16.00 ± 0.37a	7.19 ± 0.17b
	NO_3_-N (mg/kg)	373.09 ± 2.53a	262.49 ± 9.90b	361.73 ± 4.11a
	AP (mg/kg)	142.85 ± 1.68c	157.33 ± 3.24b	171.67 ± 1.17a
	AK (mg/kg)	845.43 ± 0.36a	761.67 ± 4.20b	686.33 ± 6.64c
	OM (g/kg)	32.81 ± 0.07a	28.77 ± 0.23b	26.30 ± 0.49c
	OC (g/kg)	56.570 ± 0.12a	49.600 ± 0.40b	45.340 ± 0.84c
QY (2023)	pH	7.39 ± 0.01a	7.18 ± 0.01b	7.15 ± 0.01c
	TN (g/kg)	1.68 ± 0.01c	1.81 ± 0.01b	1.84 ± 0.01a
	TP (g/kg)	1.71 ± 0.01a	1.69 ± 0.00a	1.15 ± 0.01b
	TK (g/kg)	13.35 ± 0.06a	10.65 ± 0.08b	8.87 ± 0.02c
	NH_4_-N (mg/kg)	17.14 ± 0.32a	16.67 ± 0.62a	7.53 ± 0.31b
	NO_3_-N (mg/kg)	406.43 ± 1.17a	219.16 ± 1.90b	408.40 ± 1.25a
	AP (mg/kg)	129.52 ± 2.41c	134.00 ± 1.04b	141.67 ± 1.17a
	AK (mg/kg)	545.43 ± 0.36c	658.33 ± 1.01a	579.67 ± 0.48b
	OM (g/kg)	27.71 ± 0.08a	27.44 ± 0.14a	25.63 ± 0.16b
	OC (g/kg)	47.78 ± 0.14a	47.30 ± 0.25a	44.46 ± 0.27b

TN, total nitrogen; TP, total phosphorus; TK, total potassium; AP, available phosphorus; AK, available potassium; OM, organic matter; OC, organic carbon; CK, grape monocropping; YY, grape interplanted with potato variety “Favorita”; LS, grape interplanted with potato variety “Longshu7”. The error data represent the standard error of the mean. Different letters after the data in the same row indicate significant differences among the different cropping systems according to Duncan’s multiple range test at P < 0.05.

**Figure 2:**
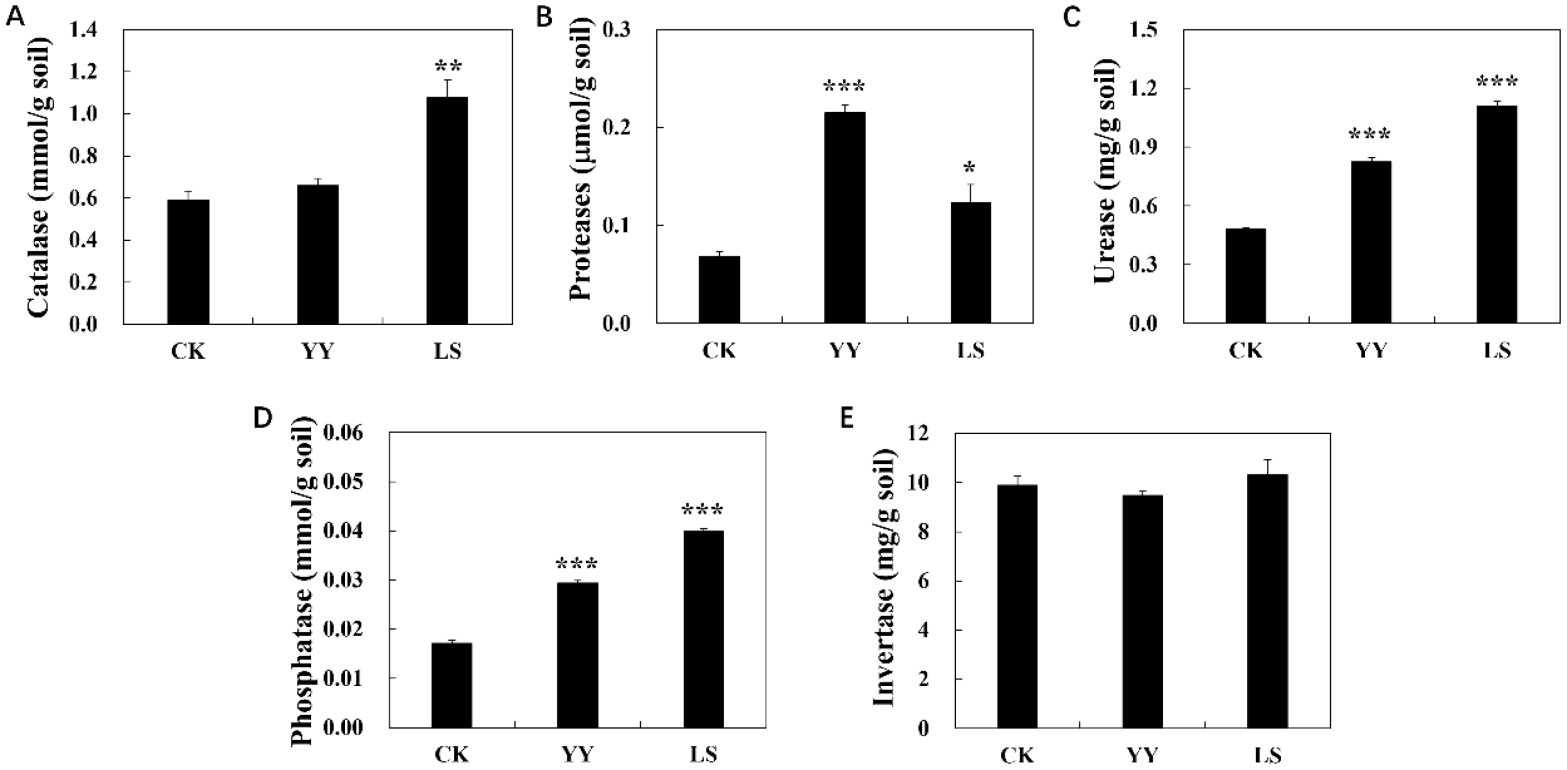
Enzyme activities of the root-zone soil in the different interplanting systems. **(A)** Catalase; **(B)** proteases; **(C)** urease; **(D)** phosphatase; **(E)** invertase. Student’s t-test was used to compare the significant differences between the interplanting and monocropping methods, and the error bars indicate the standard error of the mean with four biological replicates. No asterisk represents *P* > 0.05, * represents 0.01≤ *P* < 0.05, ** represents 0.001≤ *P <*0.01, and *** represents *P* < 0.001 for intercropping compared with monocropping.

Significant differences in soil enzyme activities were observed between the monoculture and interplanting treatments (**Figure 2**). For instance, the interplanting of YY and LS increased the acid phosphatase activity by 71.5% and 132.6%, urease activity by 71.1% and 130.3%, protease activity by 213.3% and 79.6% and catalase activity by 11.9% and 82.9%, respectively. However, the invertase activity was lower in YY and higher in LS than CK.

### 3.2 Composition and structure of the soil bacteria

Microbial community sequencing was conducted for the rhizosphere soil in different interplanting systems. Filtering and denoising of the raw sequencing data produced 516,168 high-quality sequences in 12 samples, with averages of 43,014 reads and 42,192 ASVs per sample, respectively (**Supplementary Table S1**). The dilution curves (Shannon indices) for the species in each group revealed that the bacterial diversity curves were close to the asymptote, indicating that the depth of sequencing was sufficient for all the samples (**Supplementary Figure S2**).

**Figure 3:**
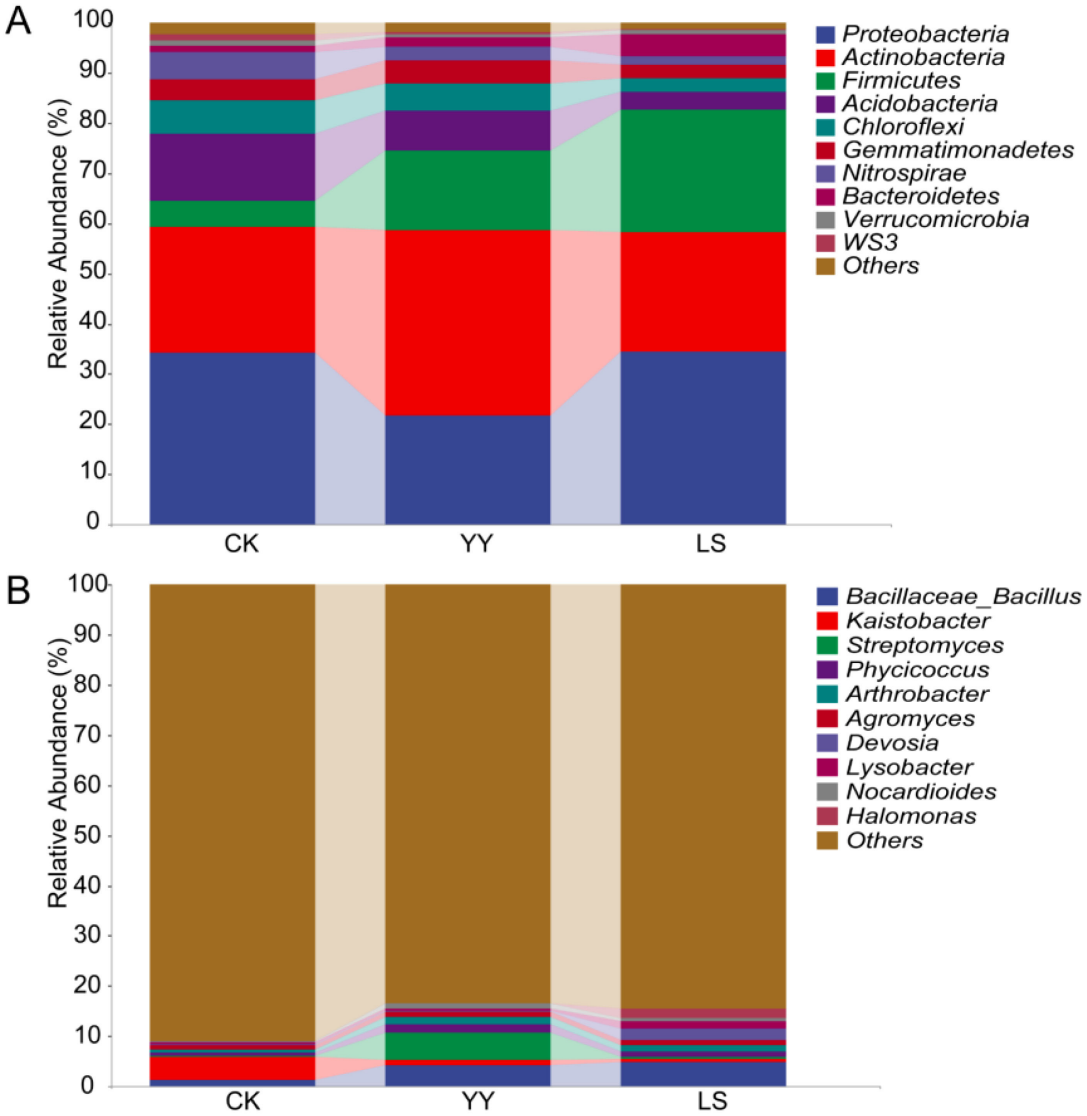
Relative abundances of the top 10 bacteria at the phylum and genus levels in the monoculture and interplanting systems. **(A)** Top 10 bacterial phyla. **(B)** Top 10 bacterial genera.

The changes in soil microorganisms in the vineyards were elucidated by analyzing the relative abundance of microbial communities in the interplanting systems. At the phylum level, *Proteobacteria* and *Actinobacteria* accounted for more than 55% of the relative abundance in the monocropping and interplanting systems (**Figure 3A**; **Supplementary Table S2**) where *Proteobacteria* was the dominant bacterial phylum in monocropping CK (34.2% of the total) and interplanting LS (34.4% of the total). In contrast, *Actinobacteria* was the dominant phylum in interplanting YY (36.8% of the total). However, the abundance of *Proteobacteria* and *Actinobacteria* remained statistically similar for the LS and CK. However, the dominant bacteria in the interplanted systems with different varieties of potatoes were significantly different. For instance, *Actinobacteria* was the dominant bacteria in the interplanted YY system, accounting for 36.9%, whereas *Proteobacteria* was the dominant bacteria, accounting for 34.4% in the interplanted LS system but accounted for only 21.8% in the interplanted YY system. Furthermore, the relative abundance of *Firmicutes* in the interplanting systems YY and LS (16.1% and 24.4% of the total, respectively) was significantly greater than that in CK (5.2% of the total).

The bacterial communities at the order level were more diverse than those at the phylum level (**Supplementary Figure S3**, **Supplementary Table S3**). *Actinomycetales* and *Gaiellales* were the dominant bacteria in the monoculture CK, accounting for 8.7% and 8.3% of the total bacteria, respectively. However, *Actinomycetales* and *Bacillales* were the dominant bacteria, accounting for 22.3% and 15.7%, and 15.4% and 24.2% of the total bacteria, respectively, whilst the abundance of *Gaiellales* significantly decreased in the interplanting system.

At the bacterial genus level, *Bacillus, Streptomyces, Phycicoccus, Arthrobacter, Devosia, Nocardioides*, and *Halomonas* were among the top 10 genera that presented significantly greater abundances in both the YY and LS interplanting systems than in the monocropping CK (**Figure 3B**; **Supplementary Table S4**). *Kaistobacter* was the dominant bacteria (4.4%) in the monoculture CK. However, in YY and LS systems, *Bacillus* was the dominant bacteria, accounting for 4.2% and 4.7%, respectively. Compared with LS, the dominant bacterium was *Streptomyces* (accounting for 5.4%) in addition to *Bacillus* in the YY interplanting system. In brief, *Bacillus, Kaistobacter*, and *Streptomyces* were the dominant bacteria in both the monoculture and the interplanting systems. Notably, *Bacillus* had a relatively high abundance and may constitute the core genus of the interplanting systems.

**Figure 4:**
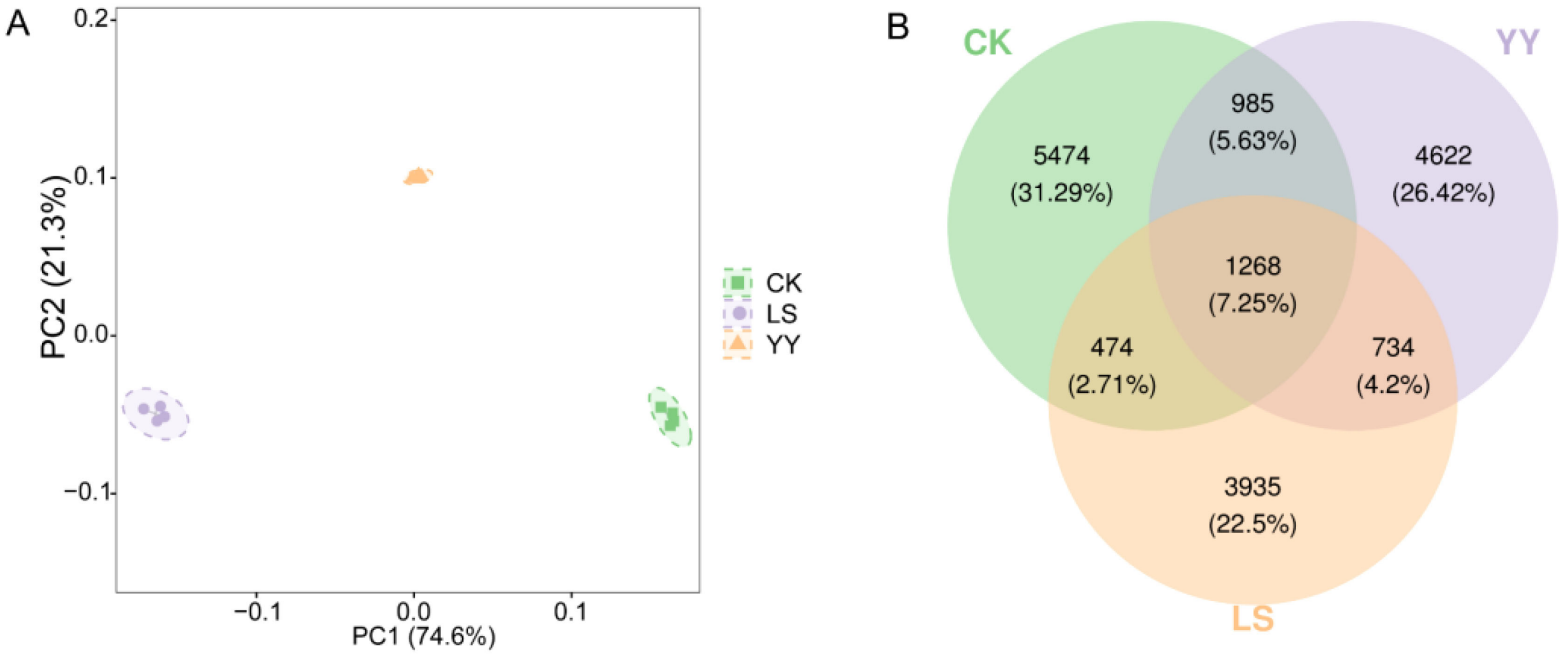
Principal coordinate analysis and Venn diagram of bacterial communities in the monoculture and interplanting systems. **(A)** Principal coordinate analysis of bacterial communities; **(B)** Venn diagram of bacterial communities in the monoculture and interplanting systems.

To further explore the differences in the effects of different interplanting systems on soil bacterial communities, principal coordinate analysis (PCoA) was conducted on the bacterial communities (**Figure 4A**). Analysis of the alpha diversity in different groups revealed significant differences in the Shannon and Simpson indices in the LS interplanting pattern compared with CK. Additionally, both the YY and LS interplanting patterns showed evolution-based diversity in terms of the PD index (**Supplementary Figure S4A**). Moreover, compared with the CK, interplanting potatoes with grapes significantly increased the relative abundance of the bacterial communities (**Supplementary Figure S4B**, **Supplementary Table S5**). Venn diagrams of beta diversity revealed that the interplanting systems were distinct from each other (**Figure 4B**). A total of 17,492 ASVs were detected, including both shared and differential ASVs. Among them, the CK group had the highest percentage of unique species at 31.29%, whereas the LS group had the lowest percentage at 22.5%, indicating that interplanting exerted a discernible influence on soil microbial diversity.

### 3.3 Relative abundance of soil bacteria

**Figure 5:**
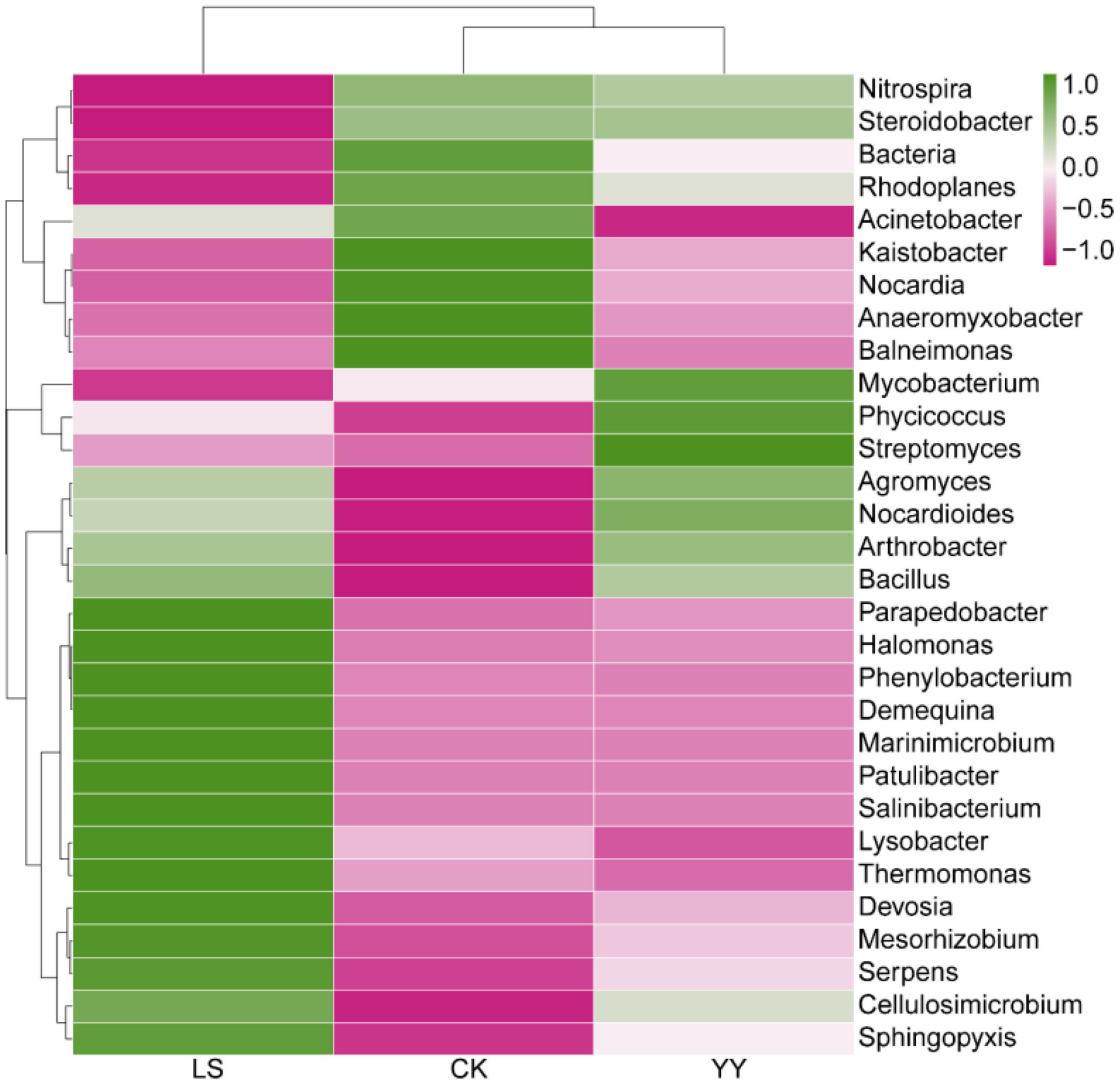
Heatmap of the relative abundance of bacteria (top 30) at the genus level in the monoculture and interplanting systems.

The distributions of the bacterial communities in the top 30 most abundant genera significantly differed among the interplanting systems (**Figure 5**). The relative abundances of *Agromyces*, *Nocardioides*, *Arthrobacter*, and *Bacillus* were significantly greater in both the YY and LS interplanting systems than in the monocropping CK, whereas the relative abundances of *Bacteria, Rhodoplanes, Acinetobacter, Kaistobacter, Nocardia, Anaeromyxobacter*, and *Balneimonas* were significantly lower in the intercropping systems YY and LS. In contrast, the relative abundances of *Mycobacterium*, *Phycicoccus*, and *Streptomyces* were significantly greater after interplanting YY, and the relative abundances of *Parapedobacter, Halomonas, Phenylobacterium, Demequina, Marinimicrobium, Patulibacter, Salinibacterium, Lysobacter, Thermomonas, Devosia, Mesorhizobium, Serpens*, and *Sphingopyxis* were significantly greater after interplanting LS. In addition, the relative abundances of *Parapedobacter, Halomonas, Phenylobacterium, Demequina, Marinimicrobium, Patulibacter, Salinibacterium, Lysobacter, Thermomonas, Devosia, Mesorhizobium, Serpens*, and *Sphingopyxis* were significantly greater under the LS conditions than under the YY conditions. In contrast, *Mycobacterium, Phycicoccus*, and *Streptomyces* were present in significantly greater numbers under the YY conditions than under the LS conditions. Similarly, the interplanted systems differed significantly at the phylum and order levels (**Supplementary Figure S5**).

### 3.4 Biomarker taxa and functional analysis of soil bacteria

**Figure 6:**
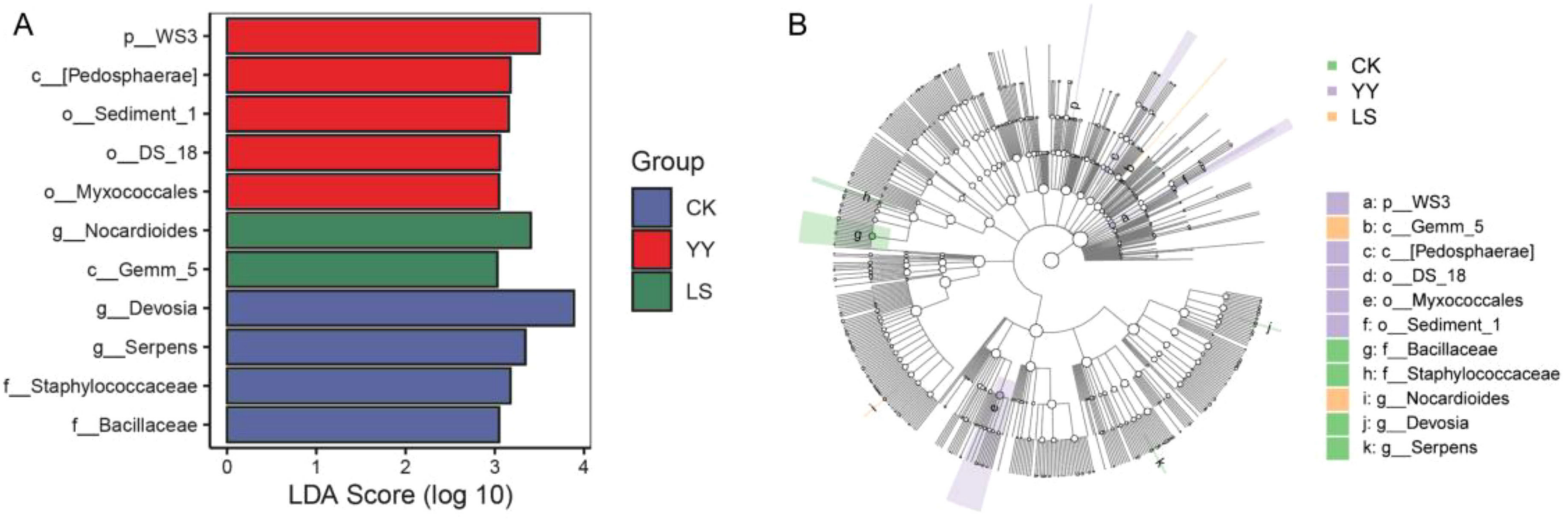
LEfSe analysis in monoculture and interplanting systems. **(A)** Histogram of LDA effect values for marker species. **(B)** Cladogram showing the different classification rank relationships between groups from phylum to genus (from the inner ring to the outer ring). When the LDA score was > 3.0, the P value was defined as *P* < 0.05.

The differences in bacterial communities at various taxonomic levels in interplanting systems were analyzed via linear discriminant effect size analysis (LEfSe) to identify marker microbial species in the intercropping systems with different potato varieties. Significant differences in bacterial species were observed between samples from different interplanting systems, as indicated by the set LDA score cutoffs (**Figure 6**). When the LDA score was > 3.0, four marker species were identified in the monoculture CK, and the numbers of marker species in the interplanting YY and LS treatments were five and two, respectively. At the genus level, *g_Devosiaa* and *g_Serpens* had the significantly highest abundances in the monoculture CK. In the interplanted LS plots, *g_Nocardioides* had the significantly highest abundance. Compared with the interplanted LS, the interplanted YY had five marker species (**Figure 6A**). Similarly, the cladogram results at the genus level revealed that *g_Devosiaa*, *g_Serpens*, and *g_Bacillaceae* were abundant in the monoculture CK. Conversely, under LS interplanting conditions, *g_Nocardioides* presented a significantly greater relative abundance. Interestingly, the YY and LS systems had different marker species (**Figure 6B**). In general, LEfSe analyses identified seven different microorganisms between the different interplanting systems.

Furthermore, KEGG functional enrichment revealed 33 major secondary subpathways in the interplanting system samples (**Supplementary Figure S6**). Among them, carbohydrate metabolism, amino acid metabolism, metabolism of cofactors and vitamins, metabolism of terpenoids and polyketides, metabolism of other amino acids, and lipid metabolism were the top six secondary subpathways in all the samples. Additionally, genetic information processing pathways, including folding, sorting, and degradation, were also found to be important (**Supplementary Table S6**).

### 3.5 Differences in metabolites

**Figure 7:**
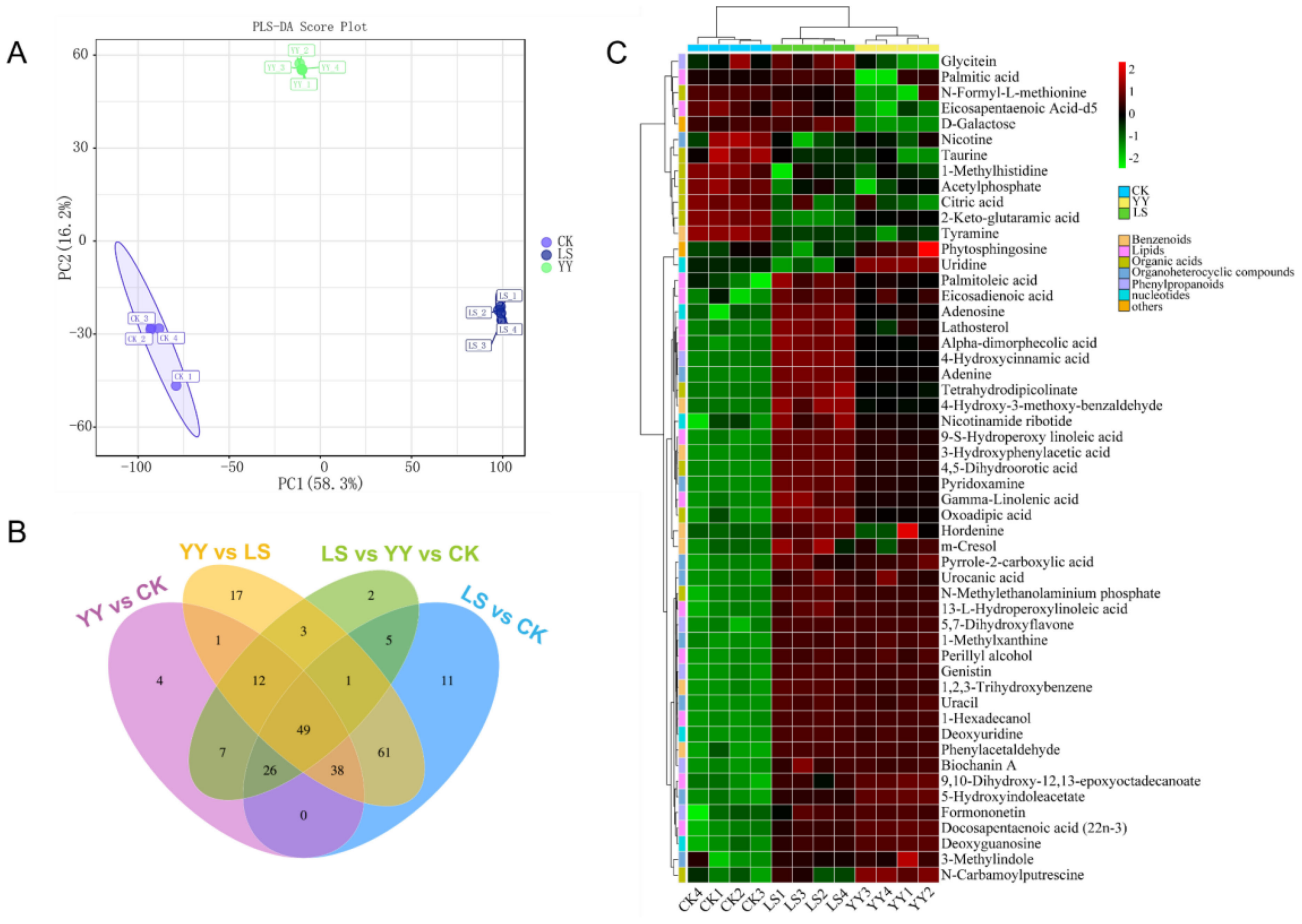
Metabolomic analysis of root-zone soil under different interplanting patterns. **(A)** PLS-DA score plots derived from metabolites; **(B)** Venn diagram of root-zone soil metabolites; **(C)** Heatmap analysis of the relative contents of differentially abundant metabolites.

To further reveal the important role of microbial communities in metabolic pathways, the PLS-DA multivariate analysis was used to investigate the variation in metabolites in root-zone soils with interplanting systems. Species accumulation and rank abundance curves demonstrated that the depth of sequencing was reliable and valid (**Supplementary Figure S7**). Score plots (Q2 was 0.996) revealed significant differences in metabolites among CK, YY, and LS (**Figure 7A**). The analysis revealed 338 differentially abundant metabolites and the interplanted YY and LS resulted in the upregulation of 106 (up to 77.3%) and 171 (up to 89.5%) differentially expressed substances, respectively (**Supplementary Figure S8**, **Supplementary Table S7**). The Venn diagrams clearly demonstrated the distinct separation of the three interplanting treatments. Among them, the YY vs LS group had the greatest number of unique substances, followed by the LS vs CK, YY vs CK, and YY vs CK groups (**Figure 7B**).

A total of 53 metabolites related to plants were identified. The identified compounds included benzenoids, lipids, nucleotides, organic acids, organoheterocyclic compounds, phenylpropanoids, and organic nitrogen compounds (**Supplementary Table S8**). In particular, lipids and organic acids exhibited the most significant changes. For instance, lipids and organic acids accounted for 24.5% (13/53) and 20.7% (11/53) of the total lipids, respectively, followed by organoheterocyclic compounds and benzenoids. The differential expression of metabolites among different interplanting systems was visualized via heatmaps. The YY interplanting was driven mainly by lipids (13-L-hydroperoxylinoleic acid, perillyl alcohol, 1-hexadecanol, 9,10-dihydroxy-12, 13-epoxyoctadecanoate, and docosapentaenoic acid (22n-3)) and organoheterocyclic compounds (pyrrole-2-carboxylic acid, urocanic acid, 1-methylxanthine, uracil, 5-hydroxyindoleacetate, and 3-methylindole). In addition, lipids (palmitoleic acid, eicosadienoic acid, lathosterol, alpha-dimorphecolic acid, 9-S-hydroperoxy linoleic acid, gamma-linolenic acid, and 13-L-hydroperoxylinoleic acid), organic acids (tetrahydrodipicolinate, 4,5-dihydroorotic acid, and oxoadipic acid), nucleotides (adenosine, nicotinamide ribotide, and deoxyuridine), phenylpropanoids (4-hydroxycinnamic acid and biochanin A), and benzenoids (phenylacetaldehyde) increased significantly in the LS treatment. However, the same metabolite drivers were not observed in the monocrop CK, interplanted YY, or interplanted LS (**Figure 7C**; **Supplementary Table S8**).

In addition, the results of the chord plot revealed that deoxyuridine (nucleotides) and uracil (organoheterocyclic compounds), which were significantly enriched in LS, were positively correlated with biochanin A (phenylpropanoids), 13-L-hydroperoxylinoleic acid (lipids), phenylacetaldehyde (benzenoids), and oxoadipic acid (organic acids) (**Figure 7C**; **Supplementary Figure S9**).

### 3.6 KEGG pathway functional annotation of differentially abundant metabolites

To understand the different pathways associated with the metabolites, analysis of metabolic pathways revealed that gamma-linolenic acid, alpha-dimorphecolic acid, 9,10-dihydroxy-12,13-epoxyoctadecanoate, 9-S-hydroperoxy linoleic acid, and 13-L-hydrodispersoleic acid are involved in the metabolism of linoleic acid (**Supplementary Figure S10A**). Citric acid, tetrahydrodipicolinate, and oxoadipic acid are involved in amino acid biosynthesis (**Supplementary Figure S10B**), whereas uracil, uridine, deoxyuridine, and 4,5-dihydroorotic acid are involved in pyrimidine metabolic processes (**Supplementary Figure S11**).

**Figure 8:**
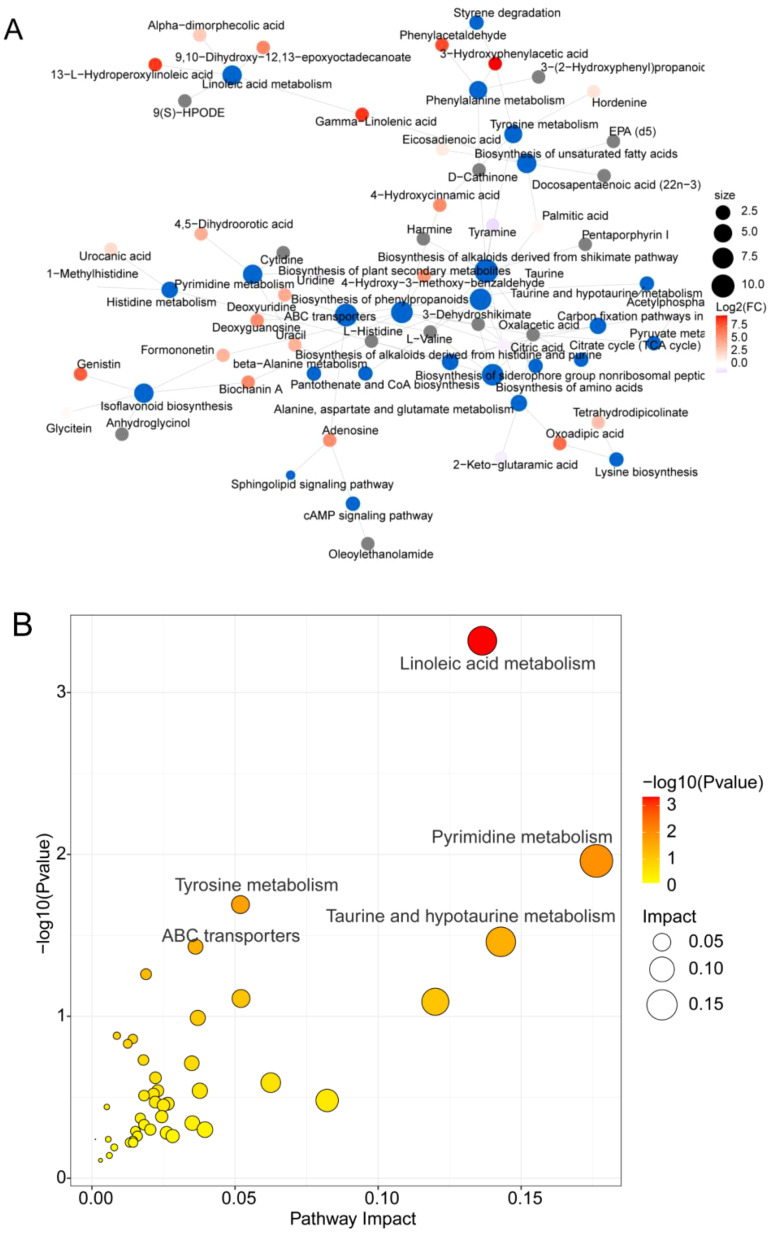
Functional analysis of metabolites involved in metabolic pathways. **(A)** Network diagram of metabolites and metabolic pathways. The blue dots indicate pathways, and the other dots indicate metabolites. The size of the pathway point indicates the number of metabolites connected to it in proportion to the number of metabolites, and the metabolite points indicate the magnitude of the log_2_(FC) value by the gradient color. **(B)** Bubble diagram of metabolic pathway-influencing factors. Impact represents the contribution of the metabolite, and the color correlates with the P value; a greater red color represents a smaller P value.

Interestingly, the red differentially abundant metabolites 13-L-hydroperoxylinoleic acid and 9,10-dihydroxy-12,13-epoxyoctadecanoate were observed in the network to be involved in the linoleic acid metabolism pathway, deoxyuridine was simultaneously involved in pyrimidine metabolism, and ABC transporters, phenylacetaldehyde, and 3-hydroxyphenylacetic acid were involved in tyrosine metabolism, phenylalanine metabolism, and styrene metabolism. Phenylacetaldehyde and 3-hydroxyphenylacetic acid are involved in tyrosine metabolism, phenylalanine metabolism, and styrene degradation pathways. Additionally, other substances are involved in pathways such as taurine and hypotaurine metabolism (**Figure 8A**; **Supplementary Table S9**), and similar results were observed for the bubble diagram depicting factors that influence metabolic pathways (**Figure 8B**). These results suggested that 13-L-hydroperoxylinoleic acid (lipid) and oxoadipic acid (organic acid) are the key differentially abundant metabolites affected by interplanting in root-soil interactions and are positively correlated with the metabolite deoxyuridine to regulate pyrimidine metabolism and ABC transporter pathway metabolism (**Figure 8**; **Supplementary Figure S9**, **Supplementary Table S8**). Phenylacetaldehyde and oxoadipic acid generally act as factors of microbial growth to increase microbial adaptation in different environments, which is consistent with altered microbial abundance in interplanting. These findings suggest that the differentially abundant metabolites, lipids and organic acid substances in the interplanting system influenced the linoleic acid metabolic pathways and improved environmental adaptation.

### 3.7 Functional correlation analysis of the metabolome and microbiome

**Figure 9:**
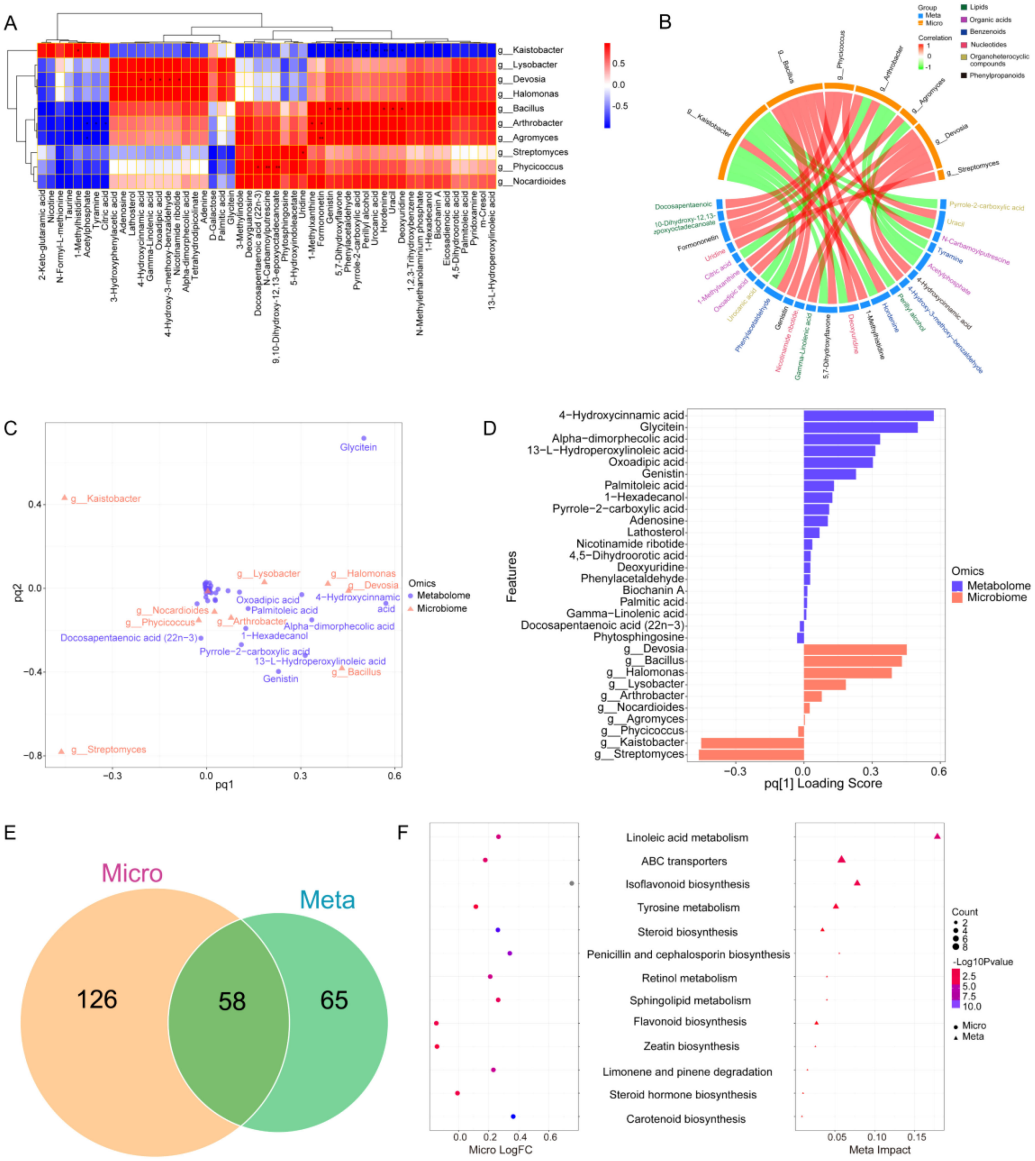
Functional correlation analysis of soil microbial taxa with soil metabolites. **(A)** Heatmap of associations between bacterial taxa and metabolites. The correlation algorithm was performed via Pearson’s algorithm, and the screening P value was 0.8. P values, *: <0.05, **: <0.01. The screening R value was 0.8. **(B)** Chord diagram of differentially abundant metabolites and bacterial taxa. The red line represents positive correlations, and the green line represents negative correlations. **(C)** O2PLS load chart of microbial taxa and metabolites. **(D)** O2PLS feature importance ranking chart. **(E)** Venn diagram of differentially enriched pathways in the microbiome and metabolome. **(F)** Correlation bubble plots of enrichment pathways in the microbiome and metabolome.

Multiomics correlation analysis can elucidate the relationships between differentially abundant metabolites and differential microorganisms in soil. The correlation heatmap revealed that nine microbial communities in the top 10 genera were positively correlated with 51 differentially abundant metabolites. Among these metabolites, *g_Bacillus* and six metabolites (genistin, 5,7-dihydroxyflavone, phenylacetaldehyde, hordenine, uracil, and deoxyuridine) and *g_Devosia* and five metabolites (4-hydroxycinnamic acid, gamma-linolenic acid, oxoadipic acid, 4-hydroxy-3-methoxy-benzaldehyde, and nicotinamide ribotide) had significant correlations. In contrast, *g_Kaistobacter* had a significant negative correlation with eight metabolites (5,7-dihydroxyflavone, phenylacetaldehyde, pyrrole-2-carboxylic acid, perillyl alcohol, urocanic acid, hordenine, uracil, and deoxyuridine) with correlations > 0.8 (**Figure 9A**). The chordal diagram of the microbiome and metabolome clearly revealed the associations between the microorganisms and metabolites. For example, *g_Bacillus* and *g_Devosia* had more metabolites that were positively correlated, whereas *g_Kaistobacter* was associated with negatively correlated metabolites (**Figure 9B**). These findings were consistent with the results of the association heatmap (**Figure 9A**).

The O2PLS analysis of microbial taxa and metabolites explains the degree of association between microbes and metabolites. Five metabolites (4-hydroxycinnamic acid, glycitein, alpha-dimorphecolic acid, 13-L-hydroperoxylinoleic acid, and oxoadipic acid) and two microorganisms (*g_Devosia* and *g_Bacillus*) were strongly associated with the metabolites (**Figure 9C**). The ranking plot of O2PLS feature importance showed similar results (**Figure 9D**), which suggest that the metabolite 13-L-hydroperoxylinoleic acid and the microorganism *g_Bacillus* had a strong association in the interplanting system compared to monocropping.

To predict microbial function, functional Venn diagrams of KEGG pathways in the microbiome and metabolome revealed 126 and 65 unique pathways specific to the microbiome and metabolome, respectively, with an overlap of 58 shared pathways (**Figure 9E**). Further correlation analysis of the impact factors and differences in pathways shared by microbes and metabolomes revealed a joint influence on the linoleic acid metabolism, ABC transporter, isoflavonoid biosynthesis, and tyrosine metabolism pathways. Among these pathways, the most significant effect was observed for linoleic acid metabolism (**Figure 9F**).

### 3.8 Association analysis of soil physicochemical properties and the microbiome

**Figure 10:**
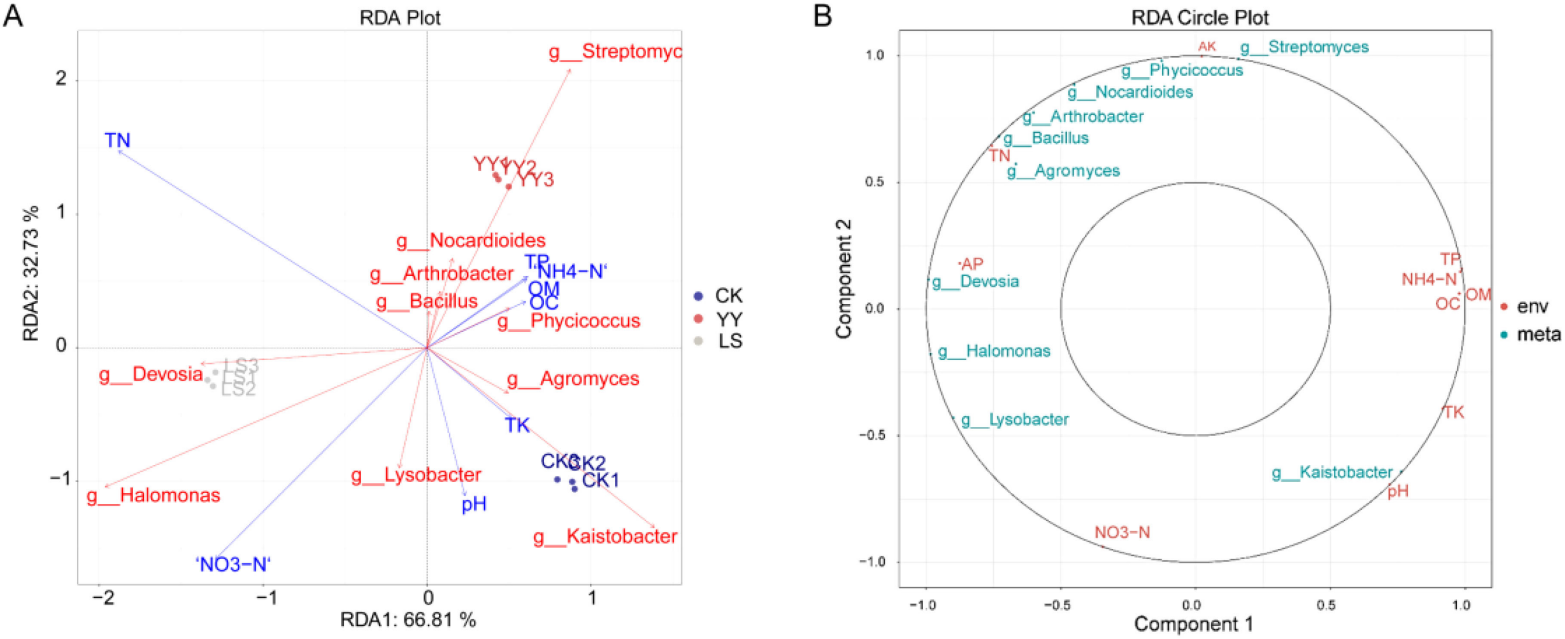
Correlation analysis of microorganisms and environmental factors. **(A)** RDA chart. Different colored dots indicate different groups of samples. The red arrows represent different microorganisms, and the blue arrows represent different environmental factors. **(B)** Correlation concentric circle diagram. The inner circle has a correlation radius of 0.5, and the outer circle has a correlation radius of 1. Red indicates environmental factors, and blue indicates different microorganisms.

The microbial distribution significantly influences the physicochemical properties of the soil, and RDA was employed to elucidate the correlation between the microorganisms and soil nutrients. The findings revealed the presence of the nearest acute angle between *g_Devosia*, *g_Bacillus* and TN, indicating that *g_Devosia* and *g_Bacillus* exert an influence on the soil TN content. Moreover, positive correlations were observed among soil TP, NH_4_-N and OM, with *g_Phycicaccus* directly impacting the soil OM content (**Figure 10A**). The concentric circle plot of correlation demonstrated that TN and *g_Bacillus* exhibited the closest proximity and the highest correlation coefficient. Similarly, strong correlations were found between *g_Devosia* and AP (**Figure 10B**).

## 4 Discussion

### 4.1 Interplanting potato with grapes increased the soil nutrient content

Compared with monocropping CK, interplanting potatoes significantly increased the contents of AP, AK, and TN (**Table 1**), indicating that interplanting potatoes can improve the soil nutrient status around the root zone of grapes, which is consistent with the results of previous studies (Wang et al., 2017; Hou et al., 2020; Li et al., 2020; Chen et al., 2023). However, differences in the range of nutrient alterations among different potato varieties were attributed to variations in their genotypes.

Grapes are perennial fruit trees, and unlike many annual crops, they are well suited to thrive in acidic soils with a pH range of 6.0–7.0 (Gretzmacher, 1970). High soil pH has been a pressing problem in vineyards in northern China. When vineyards are interplanted with potatoes, the potato root system prefers a slightly acidic environment, providing a favorable environment for the growth of grapes. In this study, our data revealed that, compared with monocropping, interplanting potatoes reduced the soil pH and promoted potato and grape growth in the most adaptable soil pH environment (Gretzmacher, 1970). Moreover, interplanting also reduced the content of OM and OC in the soil because interplanting altered the microbial community structure and increased the relative abundance of microbes (**Figures 3**, **10**; **Supplementary Figure S3**), and microbial growth consumes soil organic matter and organic carbon (Tang et al., 2022; Duan et al., 2023). Interplanting potatoes significantly increased the soil TN content because of potato invasion, which provided nutrients needed by the microbial community and increased the abundance of *Proteobacteria*, *Bacillus megaterium*, *g_Bacillus*, and nitrogen-fixing bacteria to increase the soil TN content (**Table 1**, **Figures 3**, **10**) compared with that of the monoplanted grape. Interplanting potatoes significantly decreased the contents of NH_4_-N and NO_3_-N, which was due to the different patterns of nitrogen nutrient consumption caused by growing potato plants of different genotypes (Rahav et al., 2016; Li et al., 2019).

In contrast, the TP and TK contents in the soil in the interplanting system were significantly reduced, indicating that potato plants absorbed phosphorus and potassium nutrients, with a particularly high demand for potassium. Conversely, the contents of AP and AK increased significantly in the interplanted treatments (**Table 1**), probably due to the need for more available nutrients for potato growth (**Figure 1**). This activation stimulated *Bacillus* and soil enzyme activity (phosphatase and urease) to decompose and activate insoluble phosphorus and potassium nutrients in the soil, thereby increasing the contents of AP and AK (**Figures 2**, **3**). Our findings are inconsistent with the results of previous studies (Tang et al., 2022; Duan et al., 2023).

The activities of catalase, urease, and invertase reflect the microbial population and metabolism in soils, and protease and phosphatase activities are related to the availability of nitrogen and phosphorus, respectively. Grape-potato interplanting increased the acid phosphatase, urease, protease, and catalase activities and decreased invertase activity in the interplanting system, indicating that increases in the contents of TN, AP, and AK were due to the changes in soil enzyme activity (**Figure 2**, **Table 1**). Notably, catalase, an important enzyme of soil microbial metabolism, reflects the microbial activities of the soil in the grape-potato interplanting system, suggesting that changes in soil enzyme activity within interplanting systems influence the distribution of soil microbial communities. In summary, interplanting grapes and potatoes has some advantages in improving soil fertility and nutrient availability.

### 4.2 Interplanting potato alters the abundance of the microbial community in grape soil

Intercropping of different crops and fruit trees can improve the soil nutrient status and increase the diversity of the microbial community (Cuartero et al., 2022; Li et al., 2022; Wang S. et al., 2023). In the present study, the microbial abundance of *Proteobacteria*, *Actinobacteria*, *Firmicutes*, *Gemmatimonadetes*, and *Bacteroidetes* increased under different interplanting patterns, which is in agreement with the results of previous studies on microbial community structure in agricultural soils (Wang S. et al., 2023). These findings suggest that changes in planting patterns and crop varieties influence changes in the soil microbial community structure.

We also observed that the abundance of *Firmicutes* and *Proteobacteria* was the highest in the interplanting systems, especially in LS (**Figure 3A**). It is well known that there is a wide range of *Bacillus megaterium* and nitrogen-fixing bacteria in *Proteobacteria* (Rahav et al., 2016; Li et al., 2019). The results of this study revealed that the microbial community structure changes to adapt better to the growth environment. In this study, *Bacillus* and *Kaistobacter* were the dominant bacteria, which influenced the composition of the soil microorganisms in the different interplanting systems. On the basis of the results of the relative abundance of bacteria, our results support the idea that the LS interplanting system supported bacteria to help itself in terms of inorganic and organic nitrogen availability (Duran et al., 2015; Wang S. et al., 2023).

*Firmicutes* bacteria, which have thick cell walls and belong to the monocotyledonous group, tend to thrive when plant nutrients are scarce during phytotrophic processes (Zhang et al., 2016). The analysis of chemical properties in the root-zone soil revealed a significant reduction in the TP, TK, NH_4_-N, NO_3_-N, and OM contents in the interplanting treatment compared with those in the monocropping CK, which may be attributed to the significant increase in the abundance of *Firmicutes* within the rhizosphere (**Table 1**, **Figure 3**).

In addition, *Actinomycetes* were found in the highest abundance under interplanting YY and play an important role in soil nutrient cycling and organic matter degradation (Duran et al., 2015; Wang S. et al., 2023). Our results revealed that the abundance of *Actinomycetes* was greater in interplanting than in monocropped soils (**Supplementary Figure S3**). The YY interplanting showed relatively low levels of organic matter and organic carbon at the HZ site (**Table 1**), suggesting that the increased abundance of *Actinomycetes* accelerated organic matter decomposition, which in turn reduced the soil organic matter contents which corroborates with previous studies (Li et al., 2020; Sun et al., 2022).

### 4.3 Correlation of the metabolome and microbiome with soil nutrients

The heatmap and chord diagram revealed a significant correlation between *g_Bacillus* and six metabolites and between *g_Devosia* and five metabolites (**Figure 9**), suggesting that the microbiota can interact with metabolites and/or these metabolites can activate microorganisms to improve soil fertility and adapt to environmental stress (**Table 1**). Combined analysis of the shared KEGG pathways in the microbiome and metabolome revealed that 13-L-hydroperoxylinoleic acid serves as a pivotal metabolite, whereas *g_Bacillus* acts as a key microorganism and is significantly and positively correlated with the TN and AP in the soil, collectively influencing the linoleic acid metabolism pathway in the interplanting system (**Figures 9**, **10**). It is hypothesized that 13-L-hydroperoxylinoleic acid and *g_Bacillus* may interact to regulate soil nutrient status in the rhizosphere of potato. These findings align with the results obtained from the soil metabolism and nutrient analyses (**Table 1**, **Figures 5**-**7**), highlighting the crucial role of lipids (13-L-hydroperoxylinoleic acid) in enhancing soil nutrients and in adaptation to stressful environments within interplanting systems. Lipids are crucial components of the plasma membrane and their importance as essential soil metabolites for facilitating plant adaptation to abiotic stresses is widely acknowledged (Tang et al., 2022; Chen et al., 2023; Wang S. et al., 2023).

By analyzing the relationships between soil microbial communities, metabolites, and nutrient changes in intercropping systems, we suggest that 13-L-hydroperoxylinoleic acid functions as a signaling molecule in lipid metabolism. The introduction of potatoes in grape-planted soil resulted in competition for nutrients and water, which triggered stress signal transduction through the lipid metabolism pathway via 13-L-hydroperoxylinoleic acid. This initiates a response to environmental stress in plants, leading to the secretion of phosphatases by potato plants and microorganisms to activate insoluble nutrients in the soil. By sharing carbon with microorganisms, potato plants promote microbial growth to facilitate the decomposition and activation of insoluble nutrients in the soil to meet the growth requirements of grapes and potatoes. This mutualistic relationship enables two harvests per year, therefore, the 13-L-hydroperoxylinoleic acid and *g_Bacillus* are advantageous for both potato and grape crops.

## 5 Conclusion

Interplanting modified the soil nutrient content and microbial communities, leading to a significant enrichment of bacteria associated with amino acid, carbohydrate, cofactor, and lipid metabolism that were characterized by increased concentrations of benzenoids, lipids, nucleotides, organic acids, organoheterocyclic compounds, phenylpropanoids, and organic nitrogen compounds. Additionally, lipids in interplanted soils were positively correlated with *Firmicutes*. The interaction between lipids (13-L-hydroperoxylinoleic acid) and *Firmicutes* (*g_Bacillus*) increased the nutrient content in the interplanting system. Thus, interplanting potatoes with grapes improved the soil nutrient status, soil enzyme activity content, and microbial community abundance. Overall, interplanting grapes with potatoes increased potato yield with no negative effect on grapes whereas the YY interplanting remained better than LS. In summary, our findings demonstrate that intercropping grapes with the potato variety ‘*Favorita*’ was better regarding improvement in soil nutrients, soil enzyme activity, the diversity of soil bacteria, and soil metabolites without causing adverse impacts on grape production.

## Data Availability

The original contributions presented in the study are publicly available. This data can be found here: NCBI, PRJNA1150671.
