# Supplementary material for: Interplanting potato with grapes improved yield and soil nutrients by optimizing the interactions of soil microorganisms and metabolites
Source: Front Plant Sci. 2024 Sep 9;15:1404589. doi: 10.3389/fpls.2024.1404589 (PMC11416926; doi:10.3389/fpls.2024.1404589)
Supplement: Supplementary file 1 [file DataSheet1.docx]

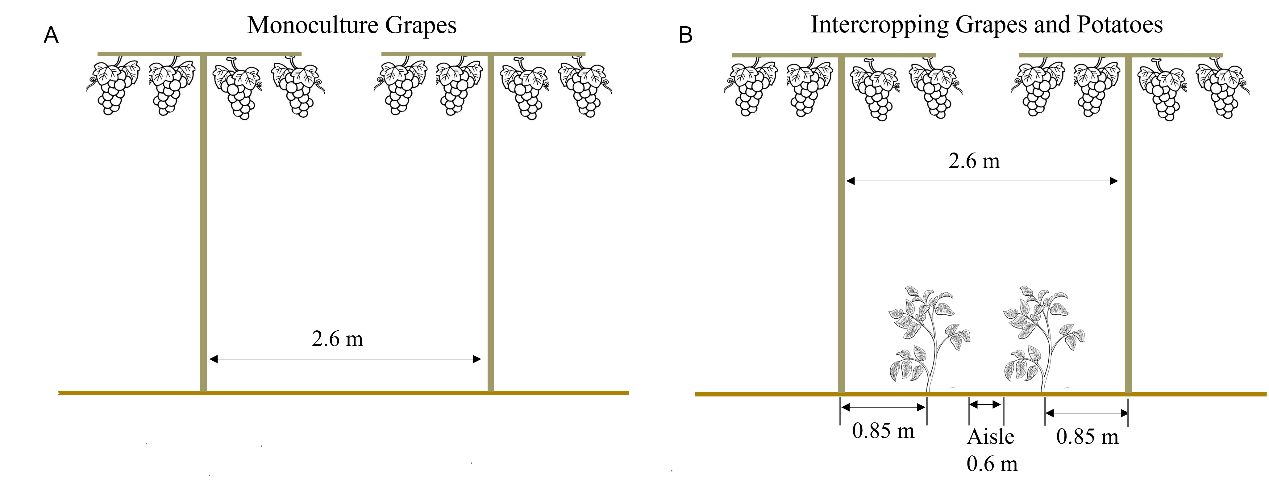


Fig. S1. Schematic diagram of interplanting potatoes within grapes. (A) monoculture grapes; (B) Interplanting potatoes within grapes.


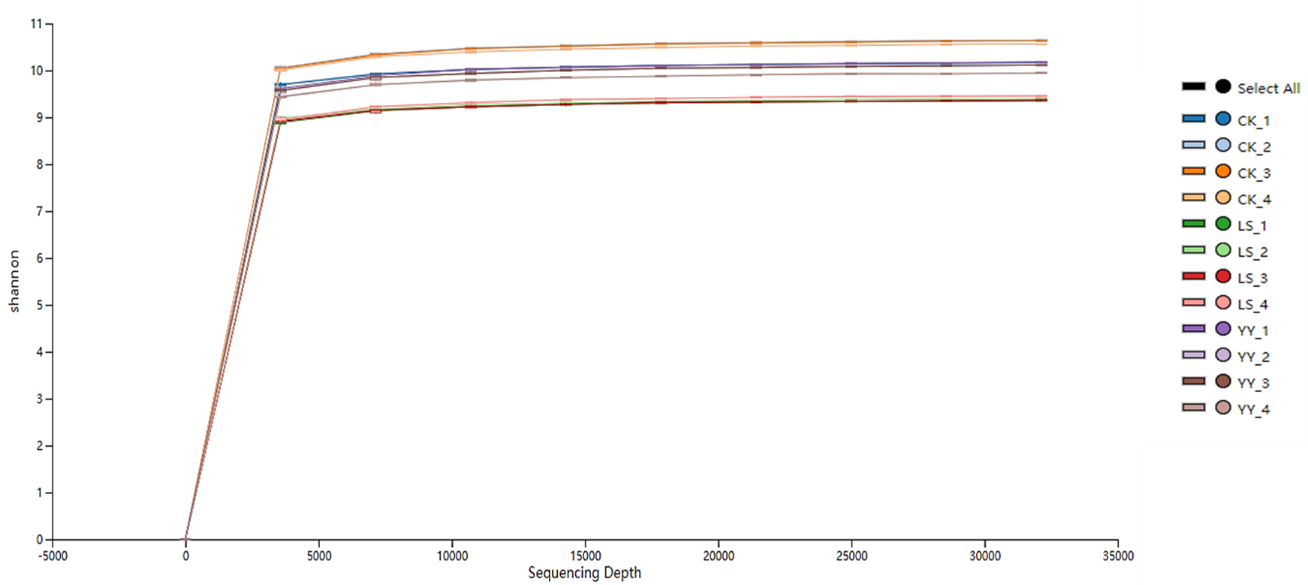


Fig. S2. Dilution curve of the bacterial community in monocropping and interplanting systems. CK, grape monocropping; YY, grape/potato (“*Favorita*”) interplanting; LS, grape/potato (“*Longshu7*”) interplanting.


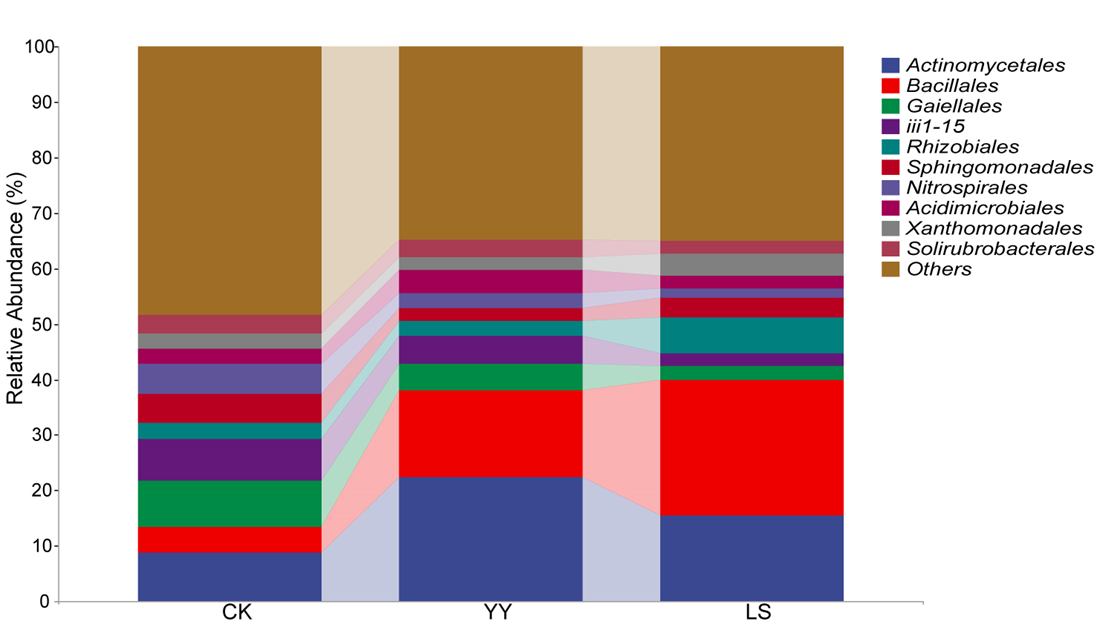


Fig. S3. Relative abundance of top10 bacterial at orders level in monoculture and interplanting systems.


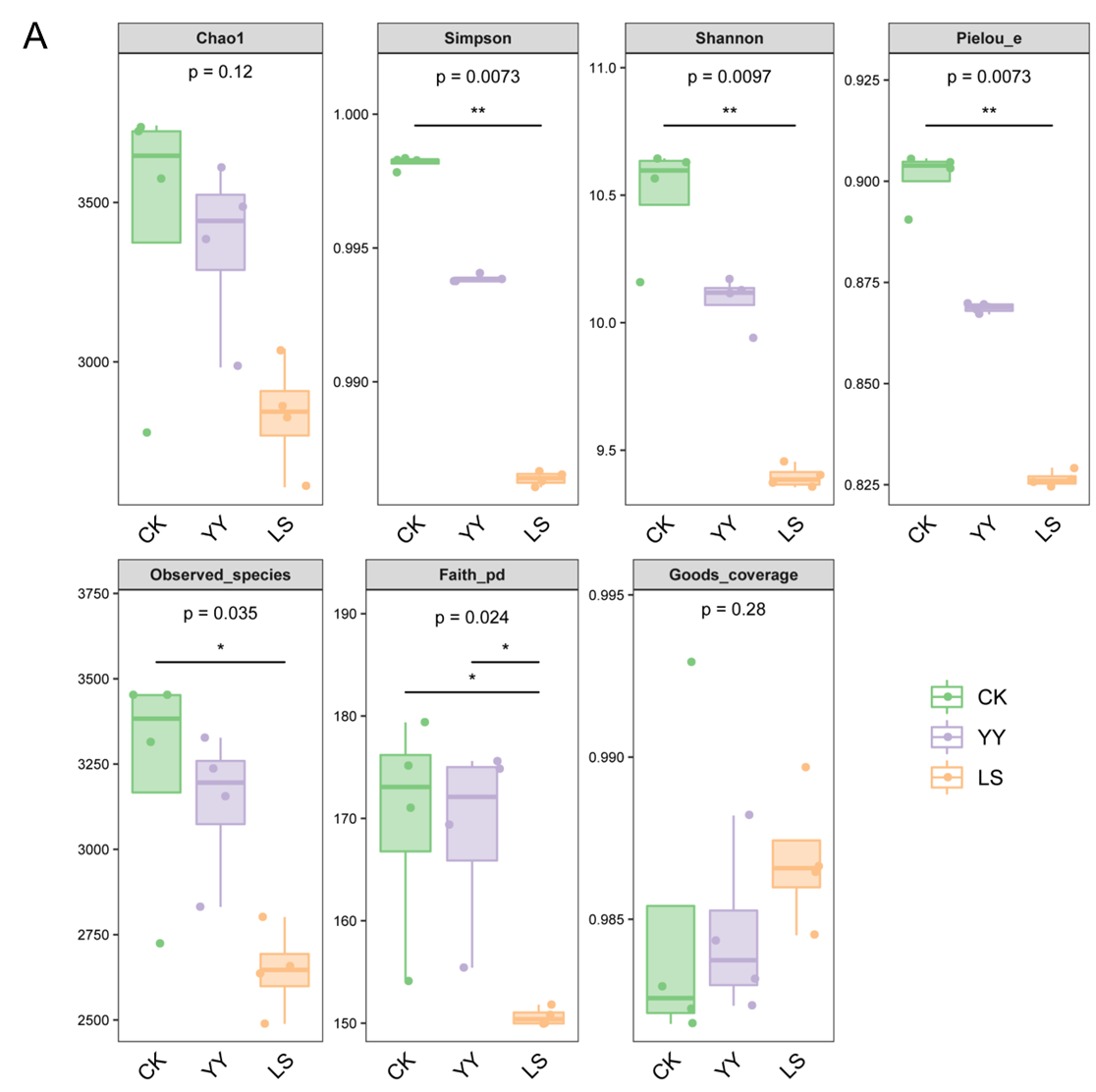


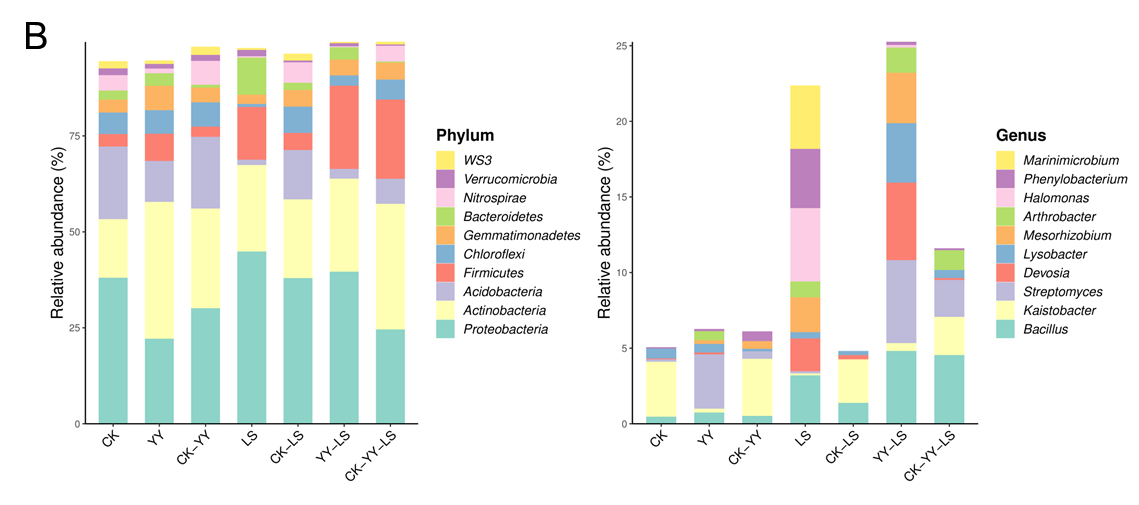


Fig. S4. Bacterial alpha-diversity and abundance composition in monocropping and interplanting systems. (A) Bacterial alpha-diversity; (B) abundance composition at the phylum and the genera.


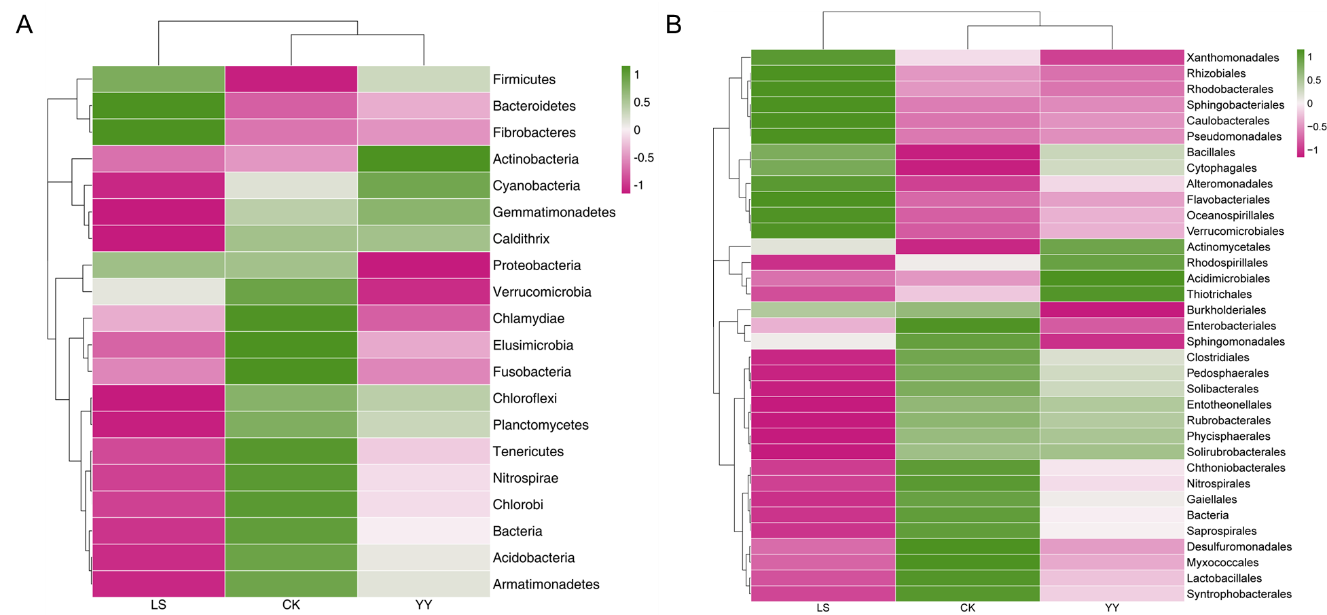


Fig. S5. Heatmap of relative abundance at the phylum and orders level in monoculture and interplanting systems. (A) Relative abundance (Top 20) at the phylum. (B) Relative abundance (Top 30) at the orders.


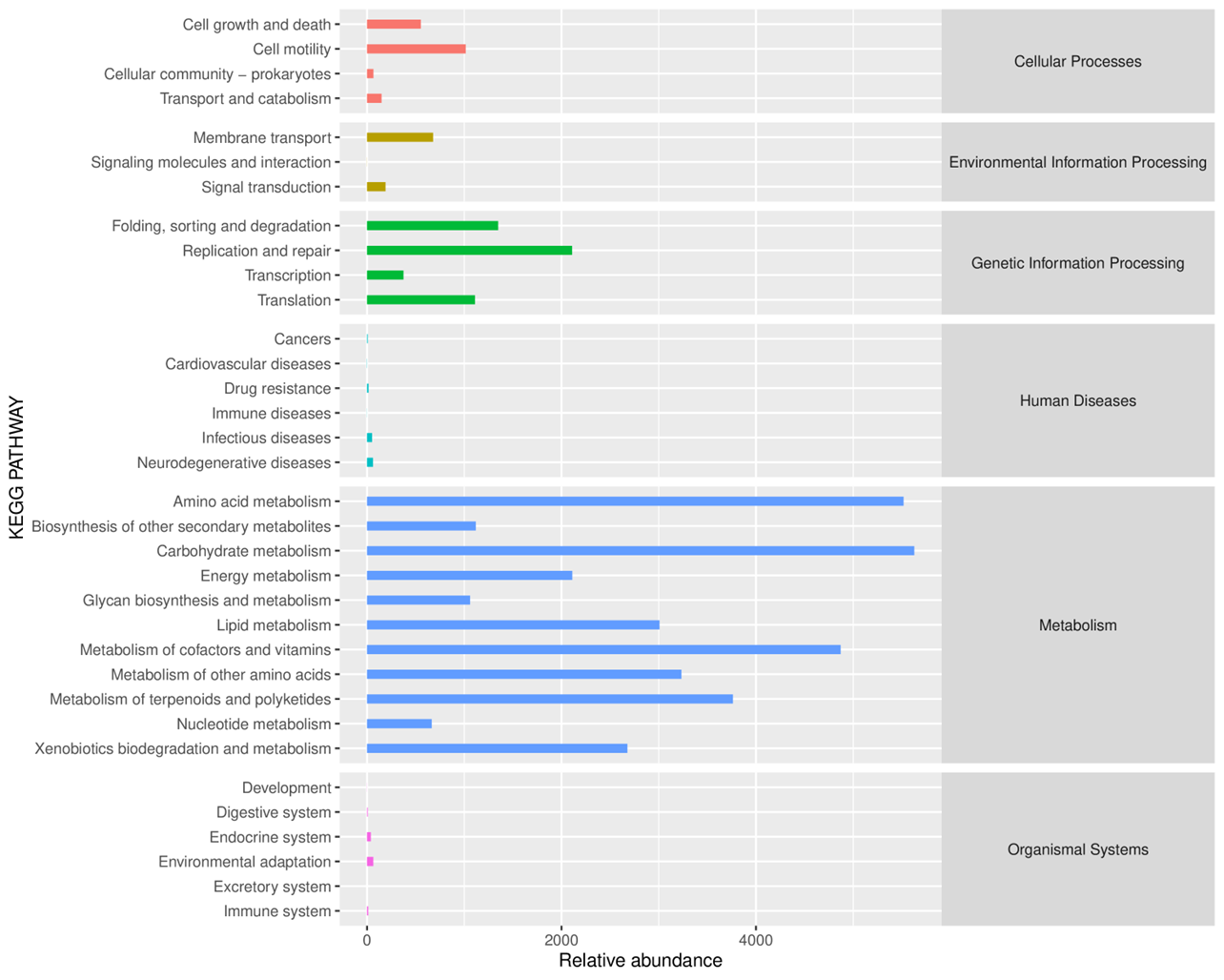


Fig. S6. Predicted abundance of KEGG secondary functional pathways.


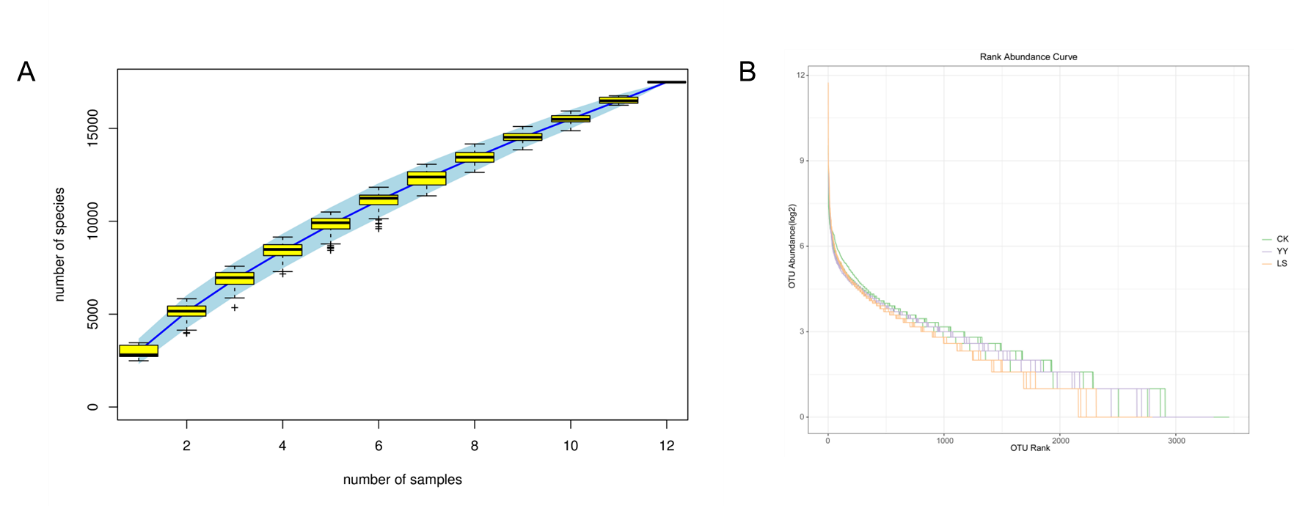


Fig. S7. Species accumulative curves and Rank abundance curves in monocropping and interplanting systems. (A) Species accumulative curves. (B) Rank abundance curves.


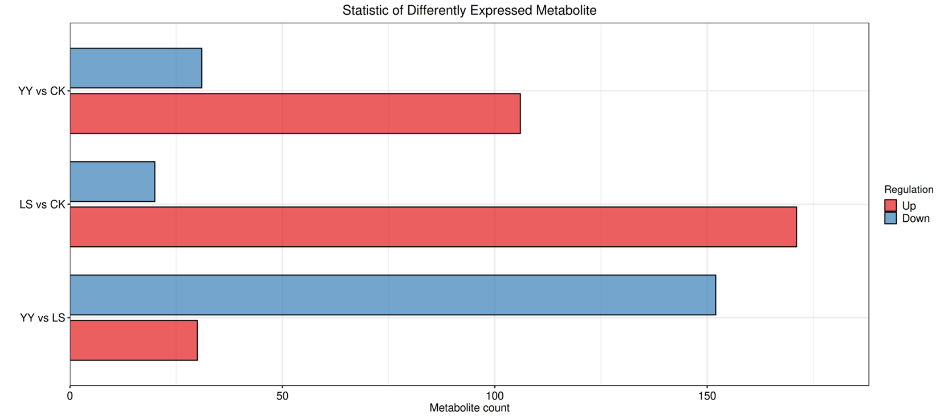


Fig. S8. Differential metabolite statistics in monoculture and interplanting systems.


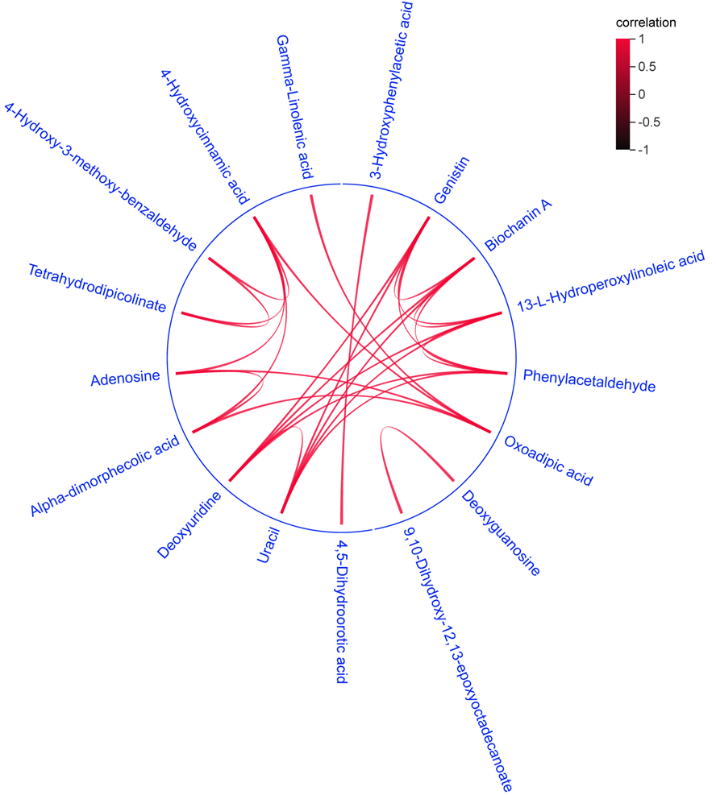


Fig. S9. Chord diagrams of different metabolites. Red lines indicate positive correlations, the black lines indicate negative correlations.


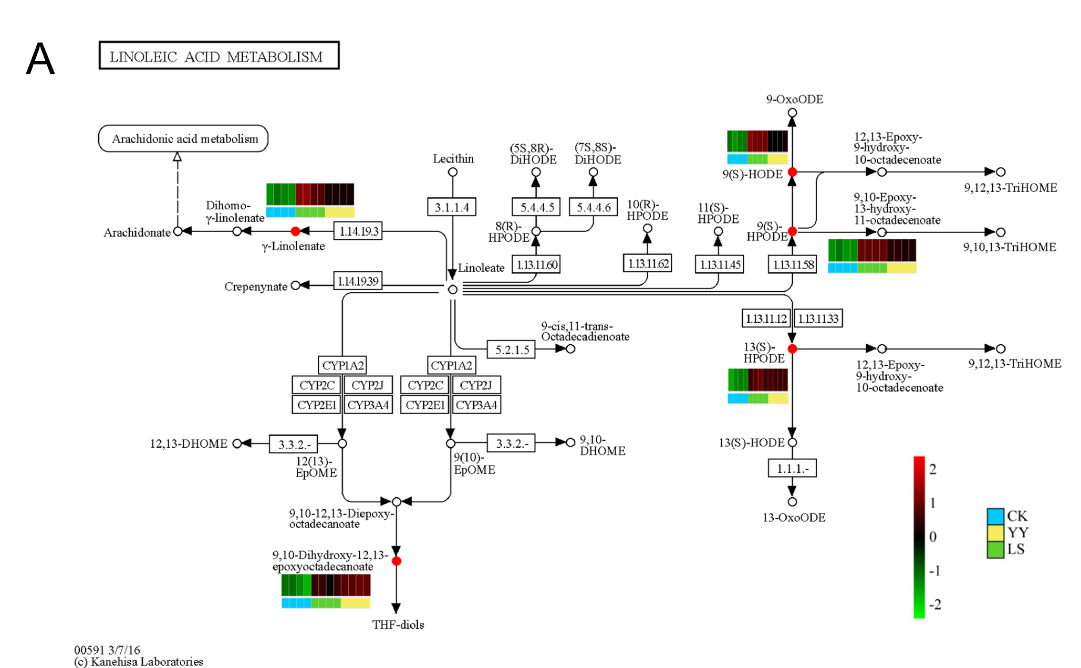

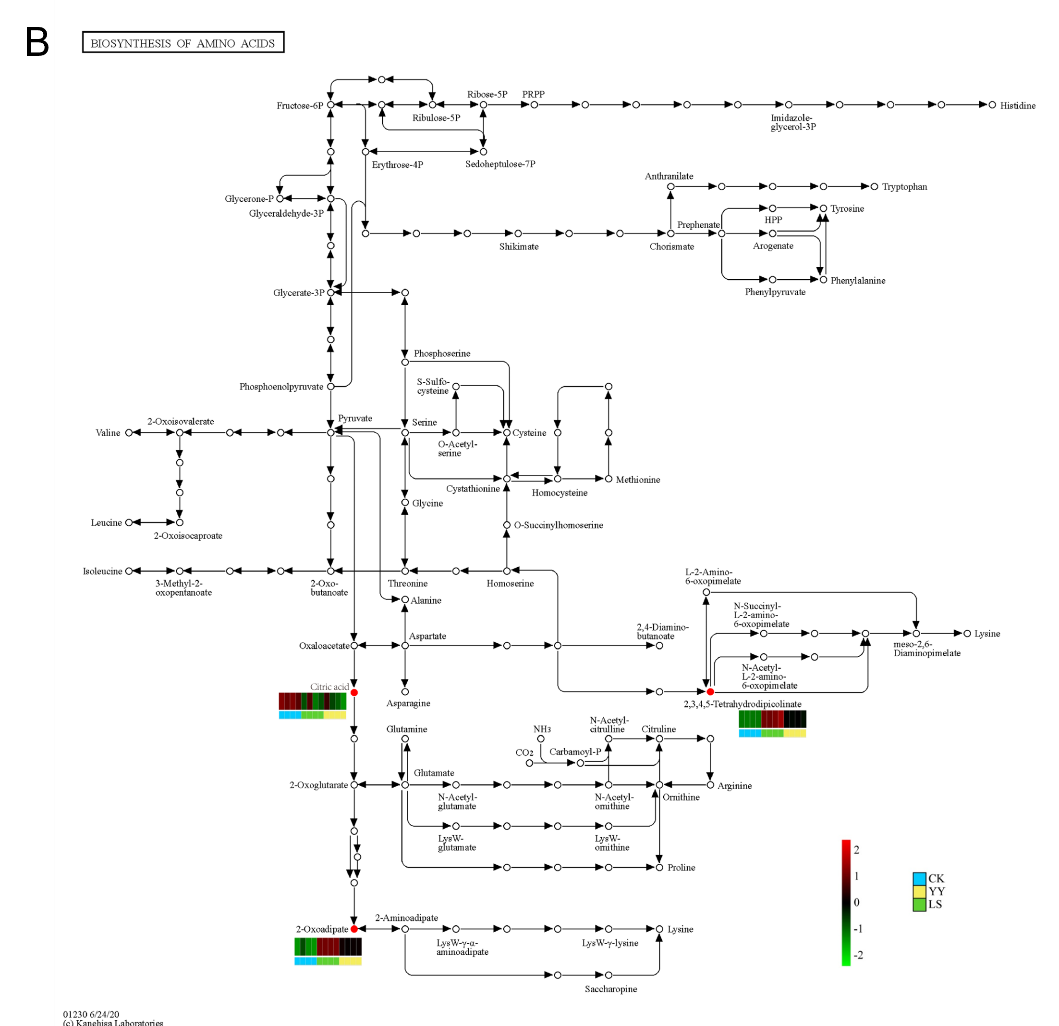


Fig. S10. Differential metabolites involved in linoleic acid metabolic pathways and amino acid biosynthesis. (A) Linoleic acid metabolic; (B) Biosynthesis of amino acid.


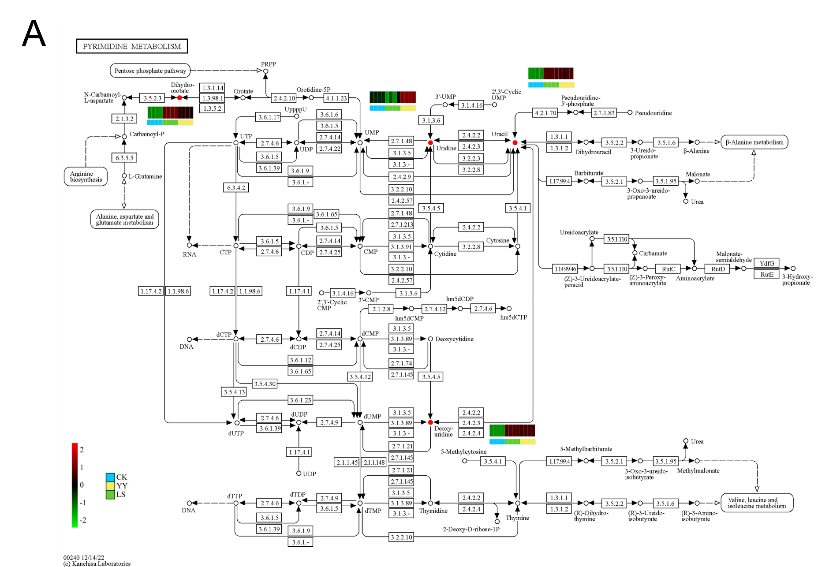


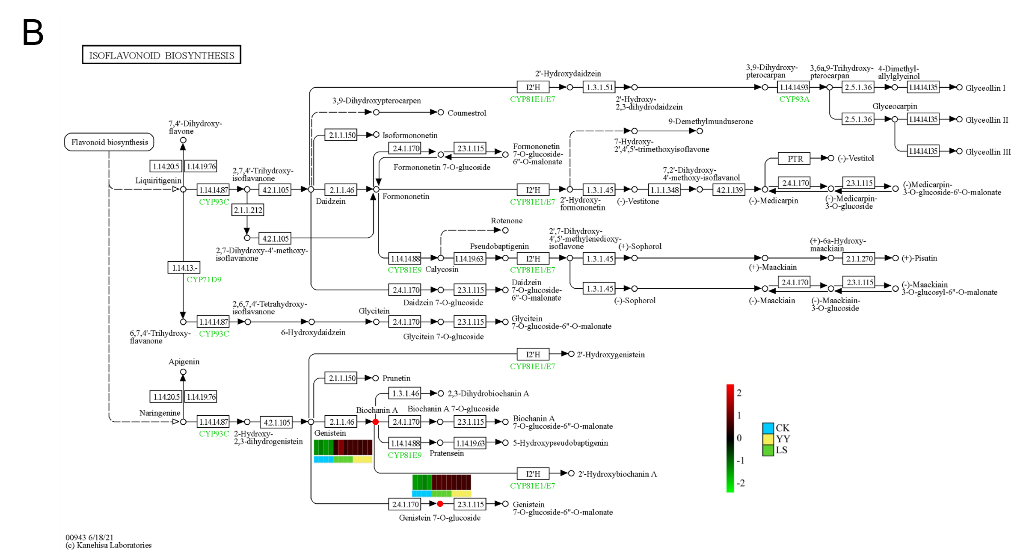


Fig. S11. Differential metabolites involved in pyrimidine metabolism and isoflavonoid biosynthetic pathways. (A) Pyrimidine metabolism; (B) Isoflavonoid biosynthesis.
